# Supplementary material for: Structural basis of nonmuscle myosin-2 autoinhibition mechanisms
Source: Nat Commun. 2026 Jun 20;17:7796. doi: 10.1038/s41467-026-74674-w (PMC13438149; doi:10.1038/s41467-026-74674-w)
Supplement: Supplementary file 2 [file 41467_2026_74674_MOESM13_ESM.pdf]

nature portfolio

Peer Review File

Structural basis of nonmuscle myosin-2 autoinhibition mechanisms

Corresponding Author: Dr Krishna Chinthalapudi

This file contains all reviewer reports in order by version, followed by all author rebuttals in order by version.

Version 0:

Reviewer comments:

Reviewer #1

(Remarks to the Author)
In this work, Heissler and coworkers explore the inhibition of NM2B, focusing on the role of motif asymmetry and of the distal region of the coiled-coil in the activation mechanism. They investigate how phosphorylations of the N-terminal extensions of the RLC can disrupt the IHM and how motif asymmetry would lead to the sequential phosphorylation of the two RLCs. They describe the flexibility of the distal part of the coiled-coil and how point mutations may contribute to the destabilization of NM2 IHM. Finally, the authors compare cardiac and NM2 IHMs, highlighting the differences existing in coiled-coil orientation.

As far as I can judge, the methodology is appropriate and the arguments are convincing. This article explores the flexibility of NM2, which allows the authors to revisit the role of the coiled-coils in this complex folding. There is no doubt that such an article will benefit the community, and I therefore recommend it for publication in Nature Communications. Most of my comments concern the presentation of the figures and minor issues or suggestions that could help readers who are not familiar with the topic. I also have three major comments/questions that should be addressed.

Major comments/questions:

-In the last part of the manuscript (p. 6, l.243-259), the authors compare the IHMs of NM2B and cardiac myosin (PDB 8Q6T), showing that the coiled-coil orientations differ. The authors chose to compare with cardiac myosin in filament, highlighting a major difference in segment 1 orientation for the cardiac IHM. However in the cardiac relaxed filament, the crowns are not strictly equivalent because the filament is pseudo-symmetric (Tamborrini et al., 2023; Dutta et al., 2023). Which crown was chosen for this comparison? Would the difference more or less pronounced by if the two other crowns were compared too? It would be of great interest to discuss this point in the paper, as cardiac myosin does not adopt exactly the same coiled-coil orientation depending on its position within the filament (Tamborrini et al., 2023; Dutta et al., 2023).

- To investigate the flexibility of the motif, the authors use a methodology referred as 3D variance analysis (3DVA) (p. 5, l. 209-220). The technique is not described in the Methods section, and the paper describing it is not cited (https://doi.org/10.1016/j.jsb.2021.107702). This information should be added. In addition, only two frames are described (1 and 20), how were these frames selected ? Was the choice based solely on the “pronounced movements of seg 1 and 3,” as stated in the text? Providing a supplementary panel or figure showing additional frames and details of the procedure, would help convince the reader.

-Have the authors checked whether a portion of the purified NM2B might be phosphorylated on the N-terminal region of the RLCs?

Minor comments:

-l. 102-103 “the motor domain cores deviate with…”. The motor domain should be defined, please indicate the residues. This term is sometimes ambiguous since it could include the Converter or not.

-l. 104-106, the relative angles of the lever arm, Supplementary Fig. 3a-c. Showing the different angles on the Supplementary figure (for example in panel 3B) would help for the understanding of the figure.

-Supp Fig. 3e: how was made the superimposition of the RLC part? On the overall motif? Only on the RLC part? Please indicate it in the legend.

-In Fig. 3 and Supp. Fig. 4-5. It is difficult to see properly Loop2 and it would help to color it distinctly. In the figures showing the cryo-EM map, the specific region of the map corresponding to Loop 2 could be highlighted or colored distinctly. This would help convince the reader that the map contains sufficient information to rebuild this flexible element.

-Supplementary Fig. 5: the actin binding region (ABR) of the BH is improperly labelled, the circle only contains the Helix-Turn-Helix (HTH) and L50 elements but not the full surface.

-I do not see how Supp Movie 5 illustrates what is written l. 187-188 and 196-197. Supplementary movie 5 seems more related to the comparison of the actin binding clefts of the motor domains of the BH, the FH and the active states (discussed with Figure 3 and in the paragraph “Mechanism underlying the reduced actin affinity of 10S”).

-Figure 4: the position of the kink and of the skip residues should be indicated, while they are named, it is not clear in the figure which is which, this would improve the understanding of the illustration.

-Figure 5: please indicate the location of the CCR on the map.

-The authors should indicate the correlation coefficients between the model and the map in supplementary table 1.

-L. 246, p.6: typo “fibersb”.

Reviewer #2

(Remarks to the Author)
Critique: This paper describes progress in the achievement of an atomic description of the 10S conformation for nonmuscle myosin II (NM2B). Non-muscle myosin II (NM2) is a ubiquitous member of the myosin superfamily and plays essential roles in regulating contractility in a wide range of non-muscle cellular processes. The transition between the autoinhibited “off” state (the folded 10S conformation) and the active “on” state (the extended 6S conformation) represents a fundamental regulatory mechanism controlling NM2 function in cells. The paper is a significant advance over previous work.

In this study, the authors report a cryo-EM single-particle structure of full-length non-muscle myosin IIB in the 10S conformation. The work provides structural insights into mechanisms that stabilize the inhibited state, including a novel heavy-chain folding arrangement and multiple interdomain interaction interfaces that prevent actin binding. The study also highlights the potential role of phosphorylation at the free head and describes distinct coiled-coil conformations across the three tail segments, emphasizing their structural flexibility.

Overall, this work contributes valuable information toward understanding the molecular basis of NM2 autoinhibition. There is a useful comparison between the NM2B structure and the corresponding structure of cardiac myosin in the native filament (PDB 8Q6T). However, several points require clarification or further discussion.

They compare their structure to the mouse cardiac myosin structure from cryoelectron tomography. Note that the structure that is being compared is at a resolution of 18Å and from a filamentous form, not a 10S form. I am unclear of the relevance since the 10S form evolved to prevent filament formation and we are comparing with a filamentous form. Moreover, they do not compare their result with the soluble HMM form of cardiac myosin (PDB 8ACT). Wouldn’t the comparison be more relevant if it compared both the HMM form and the filamentous form because then we would know at least how much of the difference is due to filaments and how much is due to the difference between muscle and non-muscle. There is also a lack of comparison with the smooth muscle 10S structure. That one should be quite similar to non-muscle. Lack of this comparison leaves more questions in the mind of the reader than the presumed clarification that is its intent.

Major items:

1. The composite reconstruction is reported at ~10 Å resolution, which is relatively low for building an accurate atomic model. While this resolution is sufficient for identifying major subdomains and interaction interfaces, it introduces uncertainty in interpreting atomic-level contacts and in mapping the precise locations of single-point mutations. Additional discussion of these limitations would strengthen the conclusions.

2. Class 4 exhibits the lowest resolution among the reported classes, despite containing a relatively large number of particles. This may suggest that structural heterogeneity or conformational variability remains within this class. It would be helpful to know whether the authors attempted further subclassification of Class 4 to explore additional conformational states. More generally, heterogeneous refinement results can depend on the number of classes provided, and this potential bias should be considered.

3. The manuscript would benefit from a clearer explanation of why Class 4 was selected to generate the composite map rather than other classes with higher reported resolution.

4. The authors use the 5.24 Å reconstruction to analyze the role of regulatory light chains (RLCs) in stabilizing the 10S conformation. It is unclear whether similar structural features are present in the other classes. Classes 2 and 3 are not discussed in detail, and the manuscript would benefit from a comparison between the models derived from maps ranging from 4.10 Å to 9.84 Å. Such comparisons could help clarify whether the observed interactions are conserved or class-specific.

5. Some important processing and validation details are not fully described in the Methods section. For example:
o the particle extraction box size used during picking. The defocus is rather large. The particle is asymmetric and if the box is too tight might have reduced their potential resolution in directions where the asymmetry of the particle caused parts to approach the edge if boxed from the uncorrected CTF images. I would have expected better resolution out of this data.
o model validation metrics
o how much of the resolution is improved after mask application and local refinement
o whether 3D classification was performed with or without image alignment

Minor comments:

Figure 1 legend. The image shown in panel A looks helical but is designated in the figure as the non-helical tail piece. The length of the coiled-coil portion of the myosin tail is pretty highly conserved, but different isoforms have different length non-helical C-termini. The Lethocerus thick filament reconstruction and the reconstruction that was built with an atomic model showed the non-helical portion in the density map. Almost nothing else has shown that. I suggest that the figure be modified to show something that is non-helical extending off of the helical part, if indeed the atomic model goes all the way to the C-terminus as implied in the figure, or give it another name. There is no density in the reconstruction for that part of the structure.

Figure 2C,D. I think that the relationship between the phosphorylated serine's of the RLC is very difficult to represent in a 2-D picture. It needs a movie. Including in the movie, a scene where the electrostatic potential before and after phosphorylation would be useful. At least one of the other movies is not particularly useful and could be replaced by a movie showing the relationship of the phosphorylated serine's and the surrounding atomic model. This is a region where the atomic model is more reliable and so should be a greater focus for the result than some of the conformational variability shown elsewhere. Not that it is not important, just that it is less important.

Figure 3. If you had to shorten the paper, I think that Figure 3 is not that important. The movie is more useful.

Figure 4. This figure is represented as the structure, when in fact is a volume representation of the atomic model. Many people will misconstrue this as the reconstruction. This should be stated in the figure legend that it is derived from the atomic model.

Figure 5D-F. Again, if these are volume representations of the atomic model, it should be so stated. If they are of the reconstruction, then the resolution cutoff needs to be stated. Probably the resolution cutoff in panels A-C should also be stated.

Figure 7. Is there not a smooth muscle atomic model available for the IHM (PDB 6XE9, 6Z47)? Why are these left out of this comparison? I should think that they are quite relevant to the conformational variation of the 10S complex. Should also point out that the comparison for cardiac muscle is from mouse and not human.

l. 175. "IMH" Shouldn't this be IHM?

l. 246. "….fibersb[47], and … ". Shouldn't this be "… fibers[47], and …"?

l. 258. "… segment 1. shows …" Shouldn't this be "… segment 1 and shows …"?

Reviewer #3

(Remarks to the Author)
I co-reviewed this manuscript with one of the reviewers who provided the listed reports. This is part of the Nature Communications initiative to facilitate training in peer review and to provide appropriate recognition for Early Career Researchers who co-review manuscripts.

Version 1:

Reviewer comments:

Reviewer #1

(Remarks to the Author)

All my comments have been satisfactorily addressed. I congratulate the authors on their excellent work.

Julien Robert-Paganin

Reviewer #2

(Remarks to the Author)
This revision is a clear improvement over the original submission. I am satisfied with it. I have only a a few minor comments given below that the authors can choose to ignore if they wish. The paper is a significant advance over their previous work and nicely presented.

Minor comments:

l. 105. The authors compare the Free Head and the PrePowerStroke state from scallop. What would be interesting is a comparison of the blocked head with the Dominguez et al. smooth muscle MDE structure. The Dominguez structure would look a lot like the blocked head and the scallop structure does not look much like the free head. The comparison could be added simply as text since the Dominguez structure does not have an RLC. I have always thought that the Dominguez structure had the blocked head conformation. Most of the literature just refers to it as “odd”, which it certainly is but it does point out that there is a conformation in smooth muscle myosin head that looks like the blocked head, but is not enforced by the other elements that are present in the interacting heads motif. I don’t know why I did not think to mention this in the original submission.

The authors might make some new friends if they worked a bit on the movies, either by slowing them down or changing a bit the annotation.

Supplementary Movie 5 could stand a little cleaning up as it starts up with an out of sequence frame.

Supplementary Movie 6 could be improved by just putting the color key in the upper right or left had corner and simply leaving it there. Since the movie is present to ostensibly compare with a closed state model, the only annotation needed is the actin-binding cleft. The ATP bound notation is just peculiar.

Supplementary Movie 7 should just leave the annotation on all the time. It is annoying that it switches on and off.

Supplementary Movie 9. Some of the annotation is on for less than a second. As long as the annotation helps with the understanding of the movie it should stay on. The Segment 2 and Segment 3 annotation just flashes in front of your eyes. Maybe slow the movie down a bit so that one can understand what the viewer is seeing.

Reviewer #3

(Remarks to the Author)
I co-reviewed this manuscript with one of the reviewers who provided the listed reports. This is part of the Nature Communications initiative to facilitate training in peer review and to provide appropriate recognition for Early Career Researchers who co-review manuscripts.

Open Access This Peer Review File is licensed under a Creative Commons Attribution 4.0 International License, which permits use, sharing, adaptation, distribution and reproduction in any medium or format, as long as you give appropriate credit to the original author(s) and the source, provide a link to the Creative Commons license, and indicate if changes were made.
In cases where reviewers are anonymous, credit should be given to 'Anonymous Referee' and the source.
The images or other third party material in this Peer Review File are included in the article's Creative Commons license, unless indicated otherwise in a credit line to the material. If material is not included in the article's Creative Commons license and your intended use is not permitted by statutory regulation or exceeds the permitted use, you will need to obtain permission directly from the copyright holder.
To view a copy of this license, visit https://creativecommons.org/licenses/by/4.0/

Reviewer #1 (Remarks to the Author):

In this work, Heissler and coworkers explore the inhibition of NM2B, focusing on the role of motif asymmetry and of the distal region of the coiled-coil in the activation mechanism. They investigate how phosphorylations of the N-terminal extensions of the RLC can disrupt the IHM and how motif asymmetry would lead to the sequential phosphorylation of the two RLCs. They describe the flexibility of the distal part of the coiled-coil and how point mutations may contribute to the destabilization of NM2 IHM. Finally, the authors compare cardiac and NM2 IHMs, highlighting the differences existing in coiled-coil orientation.

As far as I can judge, the methodology is appropriate and the arguments are convincing. This article explores the flexibility of NM2, which allows the authors to revisit the role of the coiled-coils in this complex folding. There is no doubt that such an article will benefit the community, and I therefore recommend it for publication in Nature Communications. Most of my comments concern the presentation of the figures and minor issues or suggestions that could help readers who are not familiar with the topic. I also have three major comments/questions that should be addressed.

We thank the reviewer for their positive and constructive evaluation of our work. We are pleased that the reviewer finds our methodology appropriate and our arguments convincing, and that the study's exploration of NM2 flexibility and coiled-coil function is recognized as a valuable contribution to the field. We have carefully addressed all major and minor comments. We believe these changes have strengthened both the clarity and impact of our work.

Major comments/questions:

1. In the last part of the manuscript (p. 6, l.243-259), the authors compare the IHMs of NM2B and cardiac myosin (PDB 8Q6T), showing that the coiled-coil orientations differ. The authors chose to compare with cardiac myosin in filament, highlighting a major difference in segment 1 orientation for the cardiac IHM. However in the cardiac relaxed filament, the crowns are not strictly equivalent because the filament is pseudo-symmetric (Tamborrini et al., 2023; Dutta et al., 2023). Which crown was chosen for this comparison? Would the difference more or less pronounced by if the two other crowns were compared too? It would be of great interest to discuss this point in the paper, as cardiac myosin does not adopt exactly the same coiled-coil orientation depending on its position within the filament (Tamborrini et al., 2023; Dutta et al., 2023).

Thank you for this comment. We compared our NM2B IHM structures and coiled-coil regions with the cardiac disordered crown (CrD). Although not explicitly stated in the original manuscript, our analysis included both human (PDB ID: 8G4L) and mouse (PDB ID: 8Q6T) relaxed thick filament structures. For clarity of presentation, Figure 7 initially displayed only the horizontal crown from PDB entry 8G4L. We have now updated the figure to include IHMs from all crowns (CrD, CrH, and CrT), along with a comparison of NM2B segment 1 with thick filament structures.

The corresponding text has been updated in the manuscript. It now reads as follows: "Whereas the segment 1 in NM2B lies relatively flat, the corresponding segment in cardiac myosin thick filament crowns exhibits a > 25° upward tilt, with the tilted crown (CrT) exhibiting maximum deviation. Segment 1 of the horizontal crown (CrH) of cardiac myosin-2 shows a displacement of ~40 Å in the IHM region, > 50 Å at the CCR, and > 121 Å at the distal region of segment 1. The

BH of NM2 aligns more closely with that of cardiac myosin-2 BH compared to FH and segment 1 (Fig. 7).”

2. To investigate the flexibility of the motif, the authors use a methodology referred as 3D variance analysis (3DVA) (p. 5, l. 209-220). The technique is not described in the Methods section, and the paper describing it is not cited (https://doi.org/10.1016/j.jsb.2021.107702). This information should be added. In addition, only two frames are described (1 and 20), how were these frames selected ? Was the choice based solely on the “pronounced movements of seg 1 and 3,” as stated in the text? Providing a supplementary panel or figure showing additional frames and details of the procedure, would help convince the reader.

We have now included a detailed description of the 3D Variability Analysis procedure in the Methods section and added the appropriate reference to the original publication describing this method (Punjani and Fleet, 2021; J. Struct. Biol., 213:107702).
For each principal component analyzed in the default simple mode, we generated 20 frames representing the continuum of conformational states along that component. The two frames shown in Figure 5 (frames 1 and 20) correspond to the extreme ends of the first principal component and were selected because they best illustrate the most pronounced conformational differences, particularly in segments 1 and 3. To provide a more complete view, we have now included the full set of 20 frames in Figure 1 of this rebuttal letter. This data, along with Figure 5 and Supplementary Movie 6, illustrate the continuous conformational variability revealed by 3DVA.

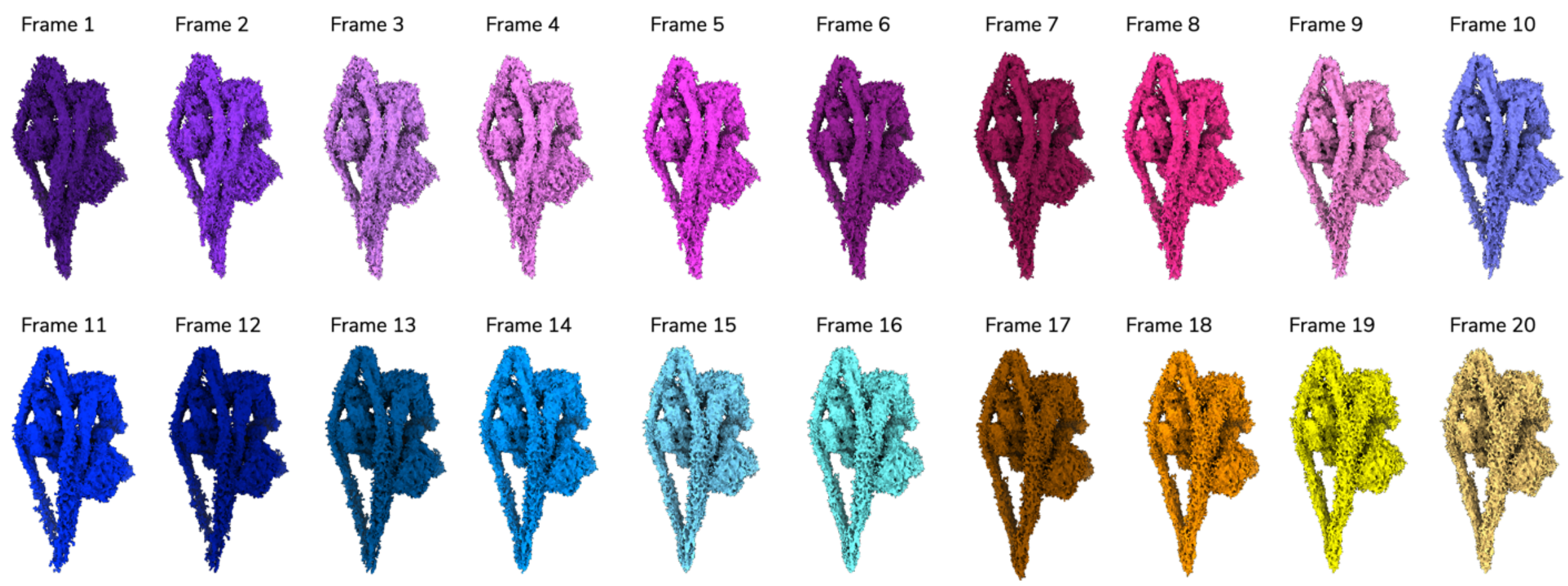

Figure 1: 3D variance analysis of the IHM of NM2B along the first principal component. 20 continuous frames are shown in distinct colors.

Have the authors checked whether a portion of the purified NM2B might be phosphorylated on the N-terminal region of the RLCs?

We thank the reviewer for this question. Full-length NM2B was recombinantly produced in the baculovirus/Sf9 insect cell system. We and others have previously shown that the RLC of myosin-2s, including nonmuscle myosin-2s, are unphosphorylated when overproduced in this system, which enables purification and downstream biochemical and structural characterization (Kovacs

et al., Biochemistry, 2004; Rovner et al., PNAS, 2008; Pollard et al., PNAS, 2017; Vasquez et al., eLife, 2016). In agreement with these reports, we found that phosphorylation increases the mass

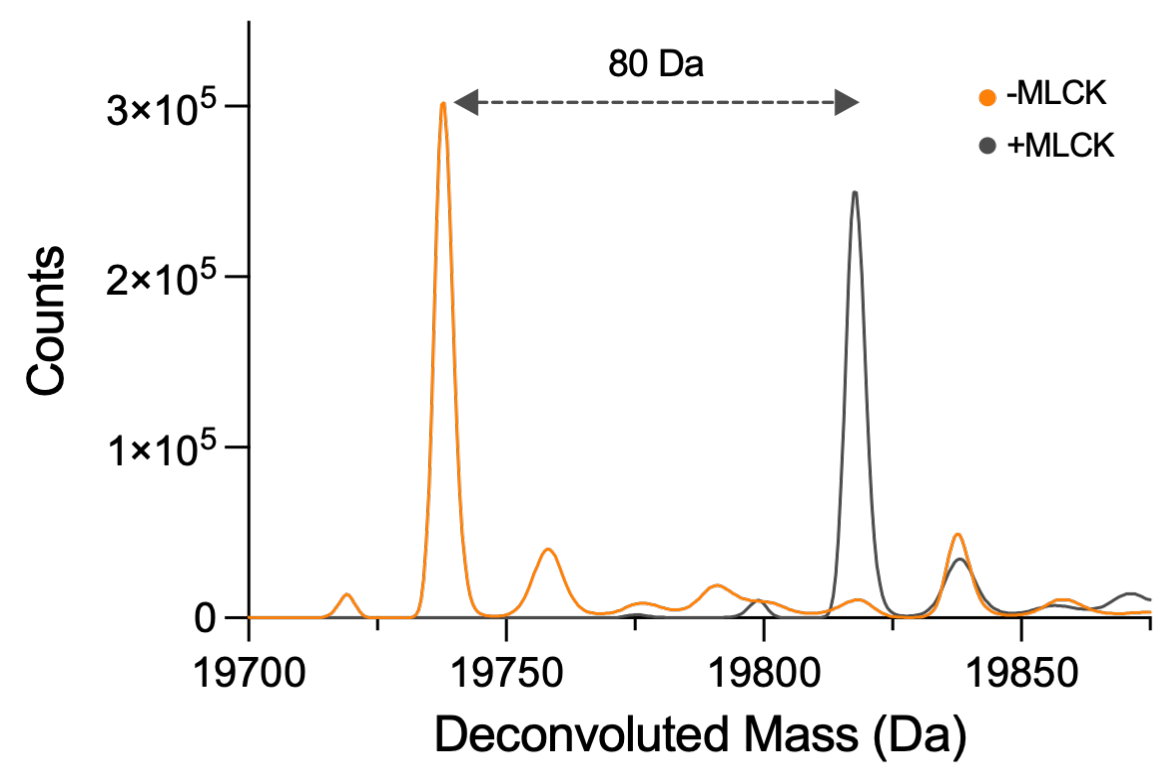

Figure 2: Intact protein mass spectrometry of NM2-bound RLC produced in the Sf9/baculovirus system (-MLCK) and after phosphorylation with MLCK (+MLCK).

of the myosin-bound RLC from 19738 Da to ~19818 Da after phosphorylation with myosin light chain kinase (MLCK), which phosphorylates S19 in the N-terminal region of the RLC (Figure 2). This is also supported by our ability to capture NM2B in the 10S state and solve its structure with cryo-EM, as phosphorylation of the regulatory light chain would instantaneously relieve autoinhibition and trigger the assembly of NM2B into filaments composed of ~30 molecules (Billington et al., JBC, 2013).

Minor comments:

1. 102-103 “the motor domain cores deviate with…”. The motor domain should be defined, please indicate the residues. This term is sometimes ambiguous since it could include the Converter or not.

We define the motor domain as residues 1-783, which includes the converter domain; this information has now been added to the Fig. 3A legend, and the converter has been labeled for clarity.

2. 104-106, the relative angles of the lever arm, Supplementary Fig. 3a-c. Showing the different angles on the Supplementary figure (for example in panel 3B) would help for the understanding of the figure.

We have now added relative lever arm angles in Supplementary Fig. 3a and 3b and also indicated them in the legend.

3. Supp Fig. 3e: how was made the superimposition of the RLC part? On the overall motif? Only on the RLC part? Please indicate it in the legend.

This superimposition was made using the distal lever arm and hook region as a reference to show the RLC position differences. This information is now included in the Supplementary Fig. 3e legend.

4. In Fig. 3 and Supp. Fig. 4-5. It is difficult to see properly Loop2 and it would help to color it distinctly. In the figures showing the cryo-EM map, the specific region of the map corresponding to Loop 2 could be highlighted or colored distinctly. This would help convince the reader that the map contains sufficient information to rebuild this flexible element.

We have revised Fig. 3A and Supplementary Figures 4 and 5 as suggested. Specifically, the loop-2 region is now highlighted in orange-red and clearly labeled. In addition, the region of the map corresponding to loop-2 was highlighted in orange for better visualization.

5. Supplementary Fig. 5: the actin binding region (ABR) of the BH is improperly labelled, the circle only contains the Helix-Turn-Helix (HTH) and L50 elements but not the full surface.

Thank you for pointing this out. This has been corrected in Supplementary Fig. 5.

6. I do not see how Supp Movie 5 illustrates what is written l. 187-188 and 196-197. Supplementary movie 5 seems more related to the comparison of the actin binding clefts of the motor domains of the BH, the FH, and the active states (discussed with Figure 3 and in the paragraph "Mechanism underlying the reduced actin affinity of 10S").

Thank you very much. We have updated it to Supplementary Movie 7.

7. Figure 4: the position of the kink and of the skip residues should be indicated, while they are named, it is not clear in the figure which is which, this would improve the understanding of the illustration.

Thank you. We have updated Figure 4 accordingly.

8. Figure 5: please indicate the location of the CCR on the map.

We now indicate the CCR in our cryo-EM map.

9. The authors should indicate the correlation coefficients between the model and the map in supplementary table 1.

We added the correlation coefficients to Supplementary Table 1.

10. L. 246, p.6: typo “fibersb”.

This typo has been corrected.

Reviewer #2 (Remarks to the Author):

Critique: This paper describes progress in the achievement of an atomic description of the 10S conformation for nonmuscle myosin II (NM2B). Non-muscle myosin II (NM2) is a ubiquitous member of the myosin superfamily and plays essential roles in regulating contractility in a wide range of non-muscle cellular processes. The transition between the autoinhibited “off” state (the folded 10S conformation) and the active “on” state (the extended 6S conformation) represents a fundamental regulatory mechanism controlling NM2 function in cells. The paper is a significant advance over previous work.
In this study, the authors report a cryo-EM single-particle structure of full-length non-muscle myosin IIB in the 10S conformation. The work provides structural insights into mechanisms that stabilize the inhibited state, including a novel heavy-chain folding arrangement and multiple interdomain interaction interfaces that prevent actin binding. The study also highlights the potential role of phosphorylation at the free head and describes distinct coiled-coil conformations across the three tail segments, emphasizing their structural flexibility.
Overall, this work contributes valuable information toward understanding the molecular basis of NM2 autoinhibition. There is a useful comparison between the NM2B structure and the corresponding structure of cardiac myosin in the native filament (PDB 8Q6T). However, several points require clarification or further discussion.
They compare their structure to the mouse cardiac myosin structure from cryoelectron tomography. Note that the structure that is being compared is at a resolution of 18Å and from a filamentous form, not a 10S form. I am unclear of the relevance since the 10S form evolved to prevent filament formation and we are comparing with a filamentous form. Moreover, they do not compare their result with the soluble HMM form of cardiac myosin (PDB 8ACT). Wouldn’t the comparison be more relevant if it compared both the HMM form and the filamentous form because then we would know at least how much of the difference is due to filaments and how much is due to the difference between muscle and non-muscle. There is also a lack of comparison with the smooth muscle 10S structure. That one should be quite similar to non-muscle. Lack of this comparison leaves more questions in the mind of the reader than the presumed clarification that is its intent.

Thank you for this comment and for recognizing that our work represents a significant advance over existing autoinhibited structures. We apologize for the mislabeling of the human cardiac thick filament PDB entry (PDB ID: 8G4L) in Figure 7. This has now been corrected in both the Discussion and the Figure 7 legend. We have also incorporated additional thick filament structures

(PDB IDs: 8Q6T and 8ACT) and updated Figure 7 accordingly; these revisions are discussed in detail in our response to Reviewer 1.

Our comparison is motivated by the fact that HMM does not include all structural segments. During manuscript preparation, we analyzed and compared our NM2B 10S structure with all available cardiac thick filament structures. Across these structures, the blocked head (BH) aligns well at the motor domain level up to the hook region. Beyond the hook, however, the IHM from HMM and the IHM from the crown regions (CrD, CrH, and CrT) of relaxed thick filaments adopt distinct conformations, none of which align well with our NM2B structure (Figure 7). Importantly, none of the available smooth muscle structures, including our 3.4 Å smooth muscle structure (PDB ID: 7MF3), resolve the coiled-coil region distal to the IHM. Some of the smooth muscle reconstructions also exhibit strong preferred orientation, leading to poor definition of the proximal tail region, which prevents a direct comparison with our NM2B structure.

As noted in the Methods, we used PDB ID: 7MF3 as a template to model the core of the IHM. In response to the reviewer's suggestion, we have now also included a Rebuttal Figure 5 comparing smooth muscle myosin IHM with our NM2B structure in this rebuttal letter.

Major comments:

1. The composite reconstruction is reported at ~10 Å resolution, which is relatively low for building an accurate atomic model. While this resolution is sufficient for identifying major subdomains and interaction interfaces, it introduces uncertainty in interpreting atomic-level contacts and in mapping the precise locations of single-point mutations. Additional discussion of these limitations would strengthen the conclusions.

Thank you very much for this comment. We have provided a detailed description in the Methods section of the manuscript explaining how the structures were built, particularly for those determined at moderate to low resolution. The relevant description from our Methods section is as follows:

"To model the IHM region, we used the high-resolution smooth muscle myosin-2 structure (PDB ID: 7MF3) as a template. Subsequent segments in the myosin tail were built by integrating structural predictions from AlphaFold ……………… Consequently, we relied on the high-resolution structure of smooth muscle myosin-2 (PDB ID: 7MF3) as a template to guide model building for all the structures solved from 4.10 Å to 9.84 Å. At these moderate to low resolutions, the side chain positioning is only approximate. The distal tail region of IHM and the adjacent tail regions exhibit substantial flexibility, intrinsically limiting the attainable resolution of the reconstructions. Accordingly, the experimental model deposited under PDB ID 9Z40 was extended using AlphaFold and coiled-coil predictions to reconstruct regions of the 10S insufficiently resolved in the cryo-EM density for confident atomic modeling."

We emphasize that this study focuses on solving the first full-length autoinhibited structure of nonmuscle myosin-2. The observed low resolution is attributed to the inherent flexibility of the tail region. Notably, we are the first to resolve structural details extending beyond 150 Å (15 nm) of the interacting-heads motif (IHM) region of either nonmuscle myosin-2 or smooth muscle myosin 2 without preferred orientation issues.

2. Class 4 exhibits the lowest resolution among the reported classes, despite containing a relatively large number of particles. This may suggest that structural heterogeneity or conformational variability remains within this class. It would be helpful to know whether the authors attempted further subclassification of Class 4 to explore additional conformational states. More generally, heterogeneous refinement results can depend on the number of classes provided, and this potential bias should be considered.

Class 4, shown in Supplementary Figure 1, comprises 13,587 particles with a global resolution of ~9.84 Å and was derived from 3D classification, following multiple rounds of heterogeneous refinement and additional 3D classifications to obtain the final particle stack. We now explicitly labeled Class 4, along with the corresponding particle number, in Supplementary Table 1. We have also revised the particle number for the full-length composite map, which was generated by combining the Class 4 IHM and Class 1 tail regions.

3. The manuscript would benefit from a clearer explanation of why Class 4 was selected to generate the composite map rather than other classes with higher reported resolution.

Thank you very much for this important comment. We selected Class 4 to generate the composite map for two main reasons.

1. Class 4 exhibits a total length of approximately 431 Å, making it the longest among the resolved classes. We have highlighted the length of each class in Supplementary Figure 1.
2. The V-shaped tail region (tail Class 1) aligns well with the extended tail observed in Class 4, showing better continuity compared to other classes.

We now have explicitly stated this in our manuscript: "The V-shaped tail segment served as a fiducial marker for reconstructing a full-length composite model of NM2B using volume maximum projection in ChimeraX. This tail region aligned well with the distal tail region of Class 4 within the V-shaped IHM. The composite structure was modeled by combining the Class 4 IHM region (~431 Å) with the V-shaped tail region from Class 1 (Supplementary Table 1 and Supplementary Figure 1)."

4. The authors use the 5.24 Å reconstruction to analyze the role of regulatory light chains (RLCs) in stabilizing the 10S conformation. It is unclear whether similar structural features are present in the other classes. Classes 2 and 3 are not discussed in detail, and the manuscript would benefit from a comparison between the models derived from maps ranging from 4.10 Å to 9.84 Å. Such comparisons could help clarify whether the observed interactions are conserved or class-specific.

Thank you very much for this comment. At the RLC level, the N-terminus of RLC_{BH} is resolved in class-1 and class-4 structures to residue K3 (Rebuttal Figure 3C, D). However, the N-terminus of RLC_{FH} is resolved only until R16. We have now included this information in the revised manuscript.

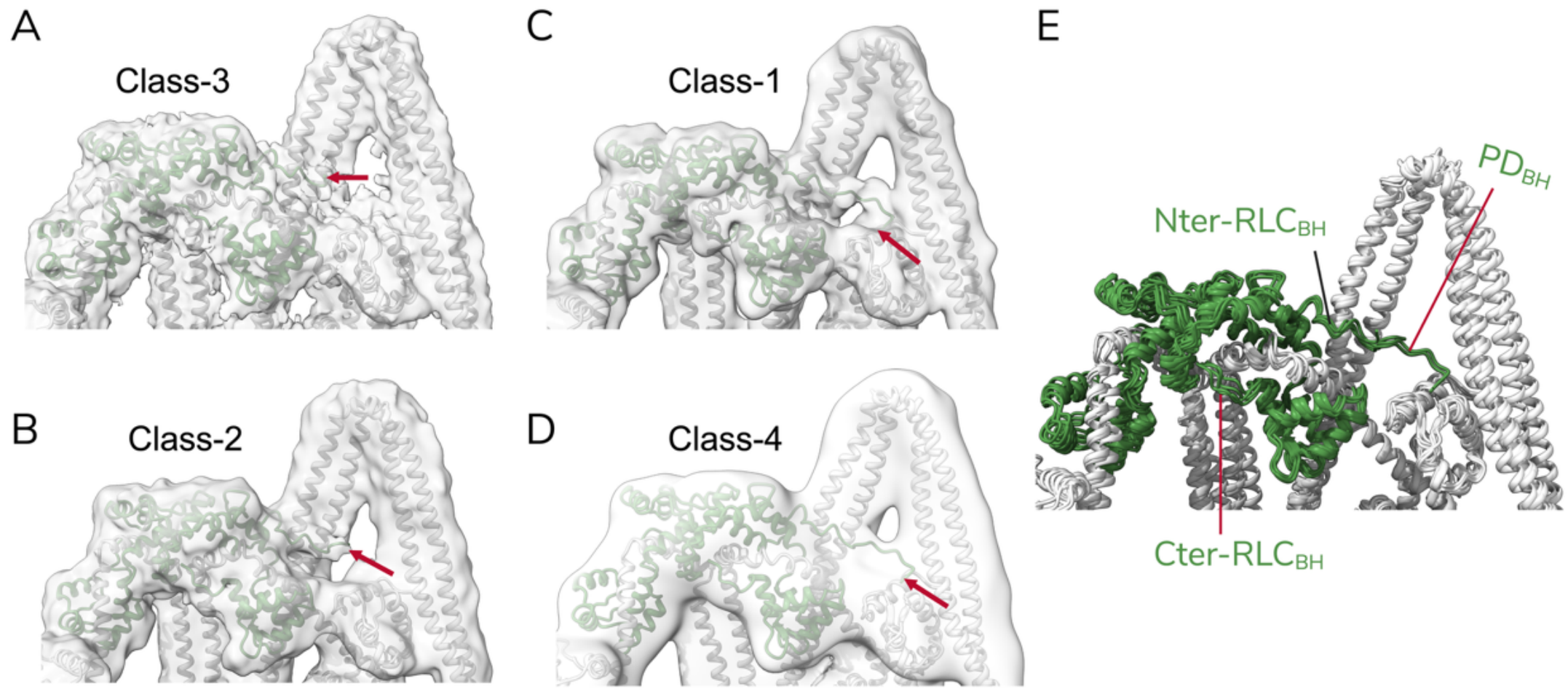

Rebuttal Figure 3: Overview of RLC-bound NM2B IHMs. (A-D) RLCs are highlighted in green across four distinct classes. The resolved N-terminus of RLC$_{BH}$ is visible in all four classes. (E) Superposition of all classes aligned at RLC$_{BH}$. Notably, only classes 1 and 4 exhibit the RLC$_{BH}$ N-terminus resolved up to K3. The red colored arrow indicates the first resolved residue in the N-terminus of RLC$_{BH}$.

5. Some important processing and validation details are not fully described in the Methods section. For example:

- the particle extraction box size used during picking. The defocus is rather large. The particle is asymmetric and if the box is too tight might have reduced their potential resolution in directions where the asymmetry of the particle caused parts to approach the edge if boxed from the uncorrected CTF images. I would have expected better resolution out of this data.

  We note that the relatively modest resolution is unlikely to arise from box size limitations but instead reflects intrinsic structural flexibility. We tested multiple particle extraction strategies, including different box sizes and 4-fold binned particles (e.g., 230 px, Rebuttal Figure 4). However, increasing the box size did not improve the resolution of the asymmetric particles, indicating that box size is not the limiting factor. We attribute this behavior to the absence of a native thick filament context for NM2 10S particles, in contrast to autoinhibited cardiac muscle myosin, where stabilizing intermolecular interactions within the filament constrain flexibility. The increased conformational flexibility, particularly in the distal tail segments, likely contributes to the observed anisotropy and limits the attainable resolution.

  We also note that the relatively large defocus values (-3.5 µm) were chosen to enhance low-frequency contrast (-1.5 to -2.5 µm) and facilitate particle identification, which is particularly important for asymmetric and low-contrast particles like 10S. While increased defocus improves image contrast, it is well established that it can attenuate high-resolution information. Thus, the defocus range used in this study represents a balance between particle visibility and preservation of the high-resolution signal and is unlikely to be the primary factor limiting resolution.

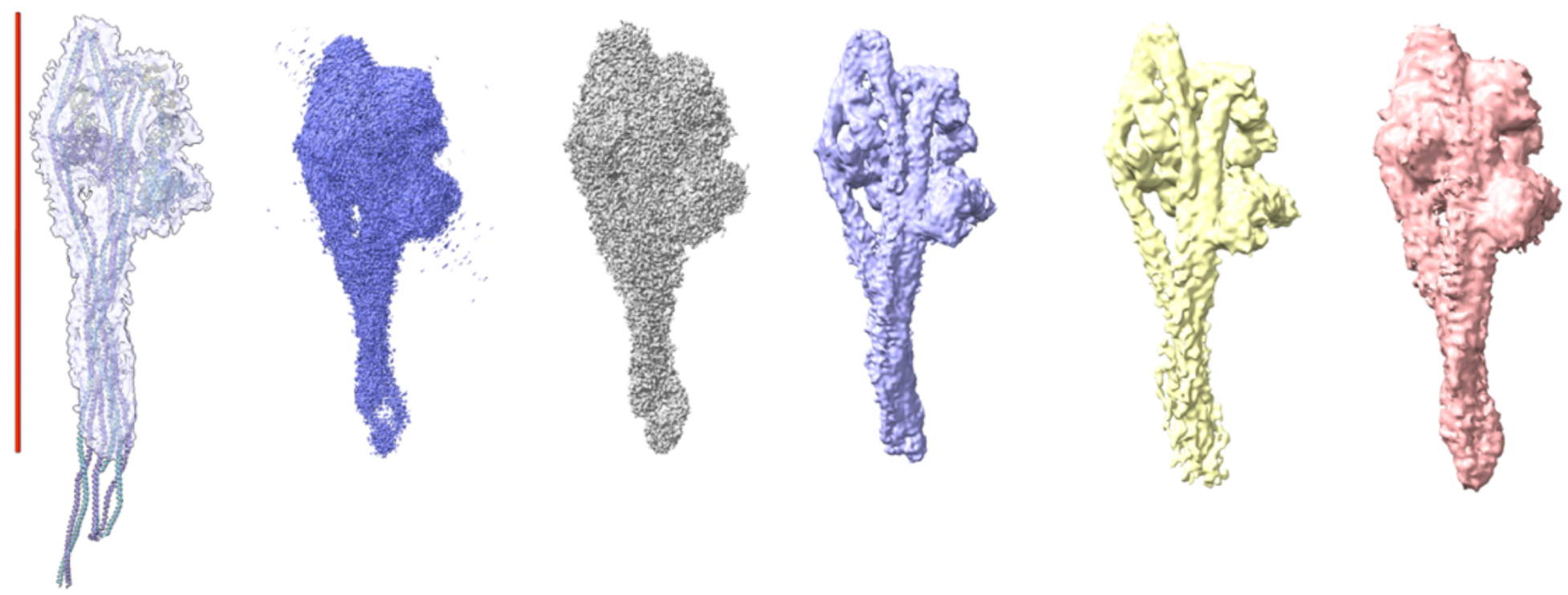

Rebuttal Figure 4: Various box sizes used to resolve the full-length NM2B structures. Length of the resolved cryo-EM density is shown.

- model validation metrics and how much of the resolution is improved after mask application and local refinement

  We provided CC_{mask} and CC_{box} for all models generated in this study, along with MolProbity and clash scores.

- whether 3D classification was performed with or without image alignment

  We also performed 3D classification without alignment first to separate structural heterogeneity while avoiding alignment bias. Subsequent refinement steps included alignment.

Minor comments:

Figure 1 legend. The image shown in panel A looks helical but is designated in the figure as the non-helical tail piece. The length of the coiled-coil portion of the myosin tail is pretty highly conserved, but different isoforms have different length non-helical C-termini. The Lethocerus thick filament reconstruction and the reconstruction that was built with an atomic model showed the non-helical portion in the density map. Almost nothing else has shown that. I suggest that the figure be modified to show something that is non-helical extending off of the helical part, if indeed the atomic model goes all the way to the C-terminus as implied in the figure, or give it another name. There is no density in the reconstruction for that part of the structure.

We thank the reviewer for identifying this mislabeling. In the original schematic, the non-helical tailpiece (NHT) region was not shown. We have now included this region and also indicated the last residue of the helical portion of the tail domain. In our structure, the helical region of segment 3 extends up to K1925, consistent with both AlphaFold predictions and available crystal structures (PDB entries 4ETO and 4CFR). Now, we show this in the revised Figure 1b.

Figure 2C,D. I think that the relationship between the phosphorylated serine's of the RLC is very difficult to represent in a 2-D picture. It needs a movie. Including in the movie, a scene where the electrostatic potential before and after phosphorylation would be useful. At least one of the other

movies is not particularly useful and could be replaced by a movie showing the relationship of the phosphorylated serine's and the surrounding atomic model. This is a region where the atomic model is more reliable and so should be a greater focus for the result than some of the conformational variability shown elsewhere. Not that it is not important, just that it is less important.

We thank the reviewer for this suggestion. In response, we have generated a new movie showing unphosphorylated S19 and phosphorylated S19 in the RLC in electrostatic surface representation to highlight the charge density distribution in the RLC_{BH} following phosphorylation. This movie has been included as Supplementary Movie 3 in the revised manuscript.

Figure 3. If you had to shorten the paper, I think that Figure 3 is not that important. The movie is more useful.

We thank the reviewer for this suggestion. We agree that the accompanying movie effectively conveys the key dynamic features of the data. We believe that Figure 3 nonetheless provides a useful static summary that facilitates interpretation of the results within the main text, particularly for readers accessing the manuscript in print or without supplemental materials. However, if space constraints necessitate shortening the manuscript, we would be happy to remove Figure 3 or move it to the Supplementary Information at the editor's discretion.

Figure 4. This figure is represented as the structure, when in fact is a volume representation of the atomic model. Many people will misconstrue this as the reconstruction. This should be stated in the figure legend that it is derived from the atomic model.

As suggested, we now explicitly state this in the figure legend.

Figure 5D-F. Again, if these are volume representations of the atomic model, it should be so stated. If they are of the reconstruction, then the resolution cutoff needs to be stated. Probably the resolution cutoff in panels A-C should also be stated.

These are cryo-EM reconstructions. We did not build atomic models for all 20 frames obtained by 3DVA analysis because of the limited particle numbers (< 5,000) present in some frames. This figure is solely intended to illustrate the degree of flexibility within the segments. We have addressed this point in the reviewer comments (Comment #2) and now report global resolution wherever applicable in Figure 5.

Figure 7. Is there not a smooth muscle atomic model available for the IHM (PDB 6XE9, 6Z47)? Why are these left out of this comparison? I should think that they are quite relevant to the conformational variation of the 10S complex. Should also point out that the comparison for cardiac muscle is from mouse and not human.

Thank you for this suggestion. We have indeed used our high-resolution structure (3.4 Å) of autoinhibited smooth muscle myosin-2 (PDB ID: 7MF3) as a template to model the NM2B IHM structures resolved in this study. Rebuttal Fig. 4 shows the comparison of our NM2B IHM structure with available smooth muscle myosin-2 IHM structures (PDB 6XE9, 6Z47). These structures

exhibit variations in the coiled-coil arrangement at bend 2; however, none of the smooth muscle myosin-2 IHM structures extend beyond the IHM region.

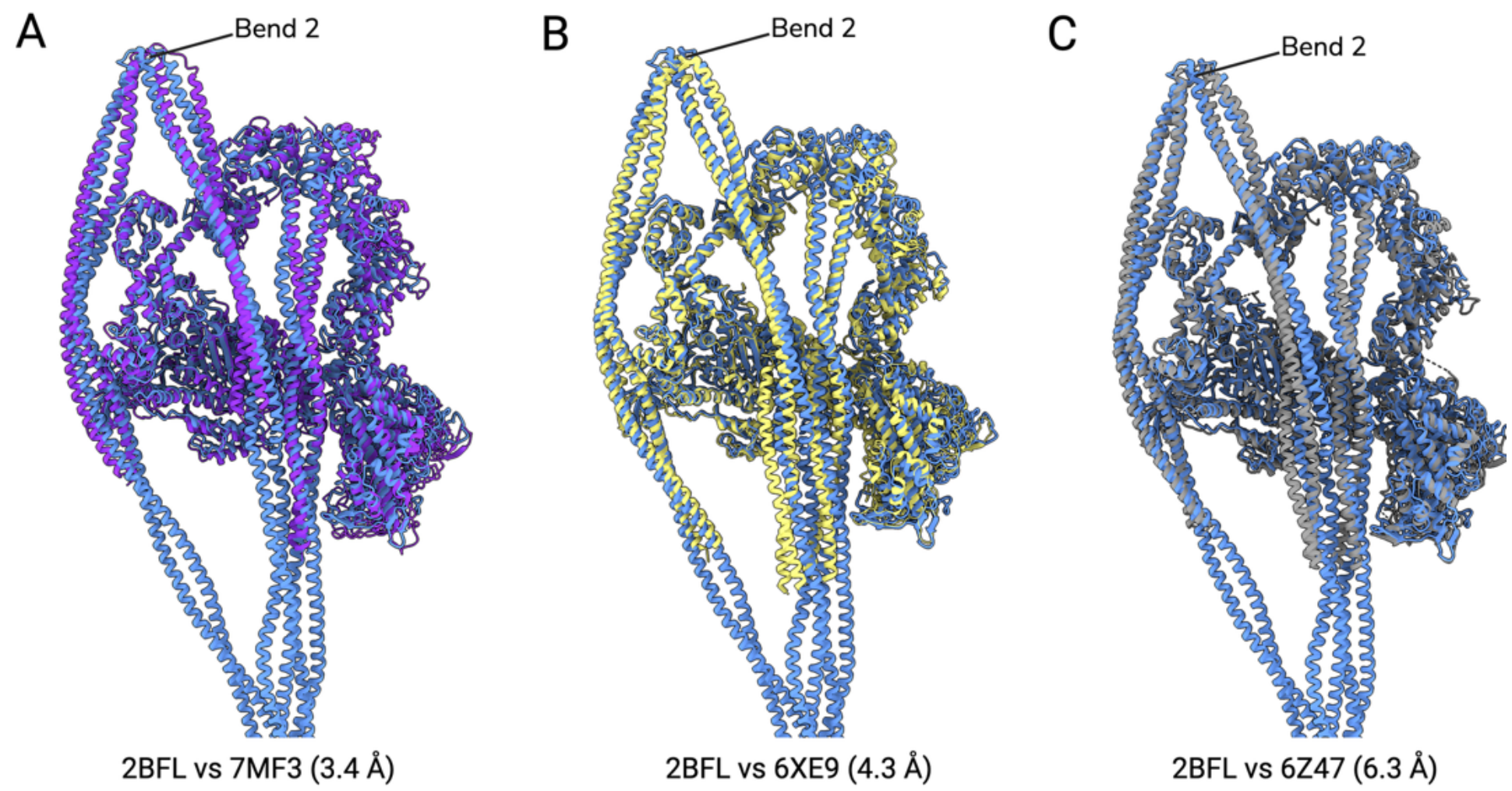

Rebuttal Figure 5: Comparison of autoinhibited NM2B structure with existing smooth muscle myosin-2 IHM structures.

We apologize for the mislabeling of the PDB entry for the human cardiac thick filament (PDB ID: 8G4L). This has now been corrected in the revised manuscript, both in the Discussion and in the legend to Figure 7.

l. 175. “IMH” Shouldn’t this be IHM?

The typo has been corrected.

l. 246. “….fibersb47, and … “. Shouldn’t this be “… fibers47, and …”?

The typo has been corrected.

l. 258. “… segment 1. shows …” Shouldn’t this be “… segment 1 and shows …”?

The typo has been corrected.

Reviewer #3 (Remarks to the Author):

I co-reviewed this manuscript with one of the reviewers who provided the listed reports. This is part of the Nature Communications initiative to facilitate training in peer review and to provide appropriate recognition for Early Career Researchers who co-review manuscripts.

Reviewer #1 (Remarks to the Author):

All my comments have been satisfactorily addressed. I congratulate the authors on their excellent work.

We thank the reviewer for their encouraging comments on our work.

Reviewer #2 (Remarks to the Author):

This revision is a clear improvement over the original submission. I am satisfied with it. I have only a few minor comments given below that the authors can choose to ignore if they wish. The paper is a significant advance over their previous work and nicely presented.

Thank you very much.

l. 105. The authors compare the Free Head and the PrePowerStroke state from scallop. What would be interesting is a comparison of the blocked head with the Dominguez et al. smooth muscle MDE structure. The Dominguez structure would look a lot like the blocked head and the scallop structure does not look much like the free head. The comparison could be added simply as text since the Dominguez structure does not have an RLC. I have always thought that the Dominguez structure had the blocked head conformation. Most of the literature just refers to it as "odd", which it certainly is but it does point out that there is a conformation in smooth muscle myosin head that looks like the blocked head, but is not enforced by the other elements that are present in the interacting heads motif. I don't know why I did not think to mention this in the original submission.

We thank the reviewer for the thoughtful suggestion. We chose to omit this analysis due to uncertainty in the RLC position and the corresponding lever-arm orientation, including the presence of a pliant region.

We now add this to our manuscript: "Structural superposition of the BH motor domain of human NM2B with the chicken smooth muscle myosin motor domain in the pre-power stroke state (PDB: 1BR4) reveals an r.m.s.d of ~1.2 Å over the aligned motor domain cores, with a relative lever arm angle deviation of ~12°.

2. The authors might make some new friends if they worked a bit on the movies, either by slowing them down or changing a bit the annotation.

Supplementary Movie 5 could stand a little cleaning up as it starts up with an out of sequence frame.

Thank you. We removed the out-of-sequence frame.

Supplementary Movie 6 could be improved by just putting the color key in the upper right or left had corner and simply leaving it there. Since the movie is present to ostensibly compare with a closed state model, the only annotation needed is the actin-binding cleft. The ATP bound notation is just peculiar.

We have added a color key and removed the remaining annotations.

Supplementary Movie 7 should just leave the annotation on all the time. It is annoying that it switches on and off.

Done.

Supplementary Movie 9. Some of the annotation is on for less than a second. As long as the annotation helps with the understanding of the movie it should stay on. The Segment 2 and Segment 3 annotation just flashes in front of your eyes. Maybe slow the movie down a bit so that one can understand what the viewer is seeing.

We slowed the movie and increased the annotation time to improve clarity.

Reviewer #3 (Remarks to the Author):

I co-reviewed this manuscript with one of the reviewers who provided the listed reports. This is part of the Nature Communications initiative to facilitate training in peer review and to provide appropriate recognition for Early Career Researchers who co-review manuscripts.

Thank you very much.
