# Supplementary material for: Structural basis of nonmuscle myosin-2 autoinhibition mechanisms
Source: Nat Commun. 2026 Jun 20;17:7796. doi: 10.1038/s41467-026-74674-w (PMC13438149; doi:10.1038/s41467-026-74674-w)

Source Data Fig. 1. Uncropped gel corresponding to Figure 1a.

Fig. 1a

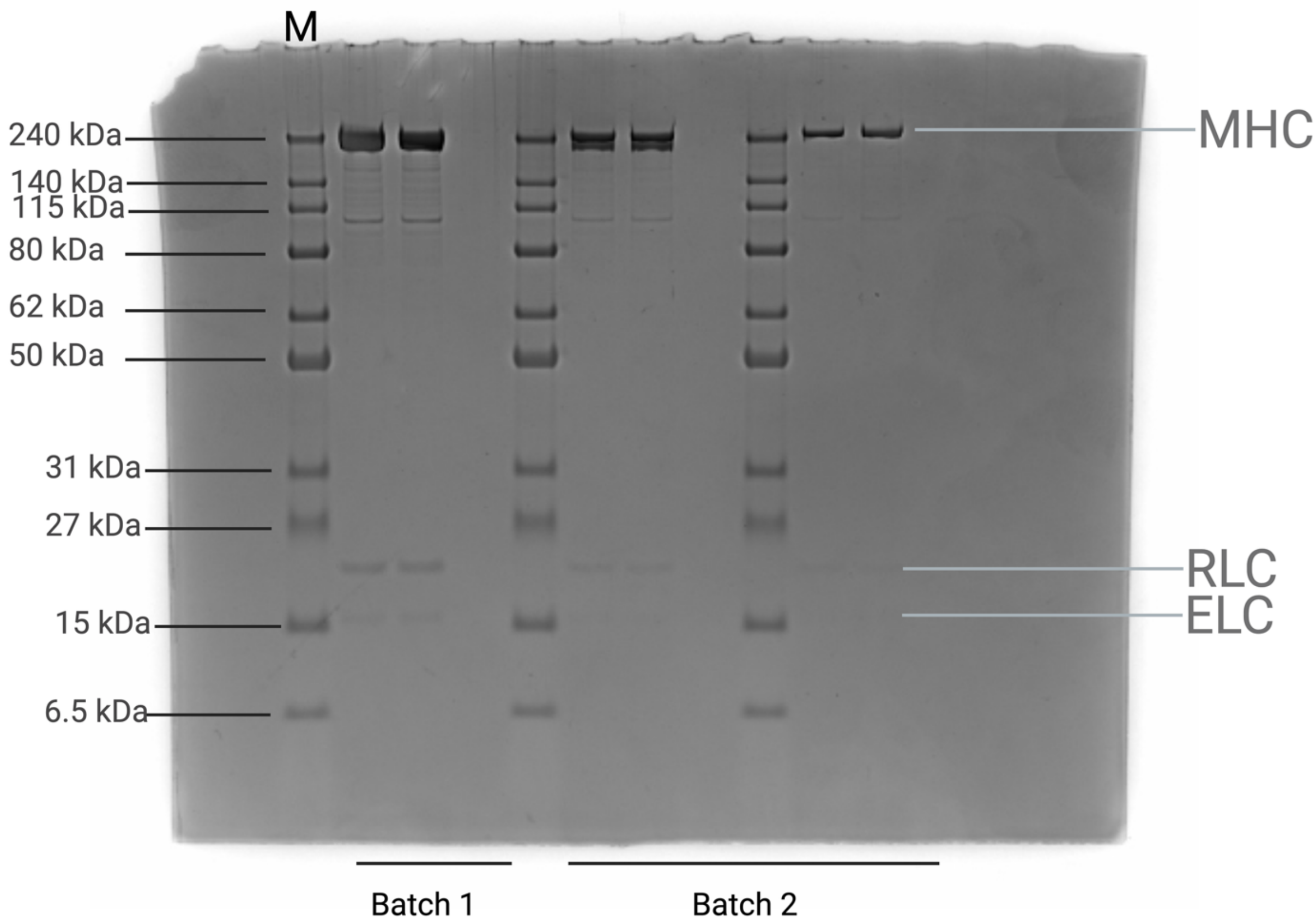

Supplement: Supplementary file 3 [file 41467_2026_74674_MOESM14_ESM.pdf]
