# Supplementary material for: Structural basis of nonmuscle myosin-2 autoinhibition mechanisms
Source: Nat Commun. 2026 Jun 20;17:7796. doi: 10.1038/s41467-026-74674-w (PMC13438149; doi:10.1038/s41467-026-74674-w)
Supplement: Supplementary file 4 [file 41467_2026_74674_MOESM1_ESM.pdf]

Structural basis of nonmuscle myosin-2 autoinhibition mechanisms

Sarah M. Heissler[1,*], Giovanna Grandinetti[1,2], James R. Sellers[3], Krishna Chinthalapudi[1,*]

Affiliations:

[1] Department of Physiology and Cell Biology, Dorothy M. Davis Heart and Lung Research Institute, The Ohio State University College of Medicine, Columbus, Ohio, USA.

[2]Center for Electron Microscopy and Analysis, College of Engineering, The Ohio State University, Columbus, OH 43210.

[3]Laboratory of Molecular Physiology, National Heart, Lung, and Blood Institute, Bethesda, Maryland, USA.

*e-mail: sarah.heissler@osumc.edu, krishna.chinthalapudi@osumc.edu

Supplementary Information

Supplementary Fig. 1	p2
Supplementary Fig. 2	p3-p4
Supplementary Fig. 3	p5
Supplementary Fig. 4	p6
Supplementary Fig. 5	p7
Supplementary Fig. 6	p8
Supplementary Fig. 7	p9
Supplementary Fig. 8	p10
Supplementary Table 1	p11
Supplementary Table 2	p12
Supplementary Movie captions	p13
Supplementary References	p14

Supplementary figures

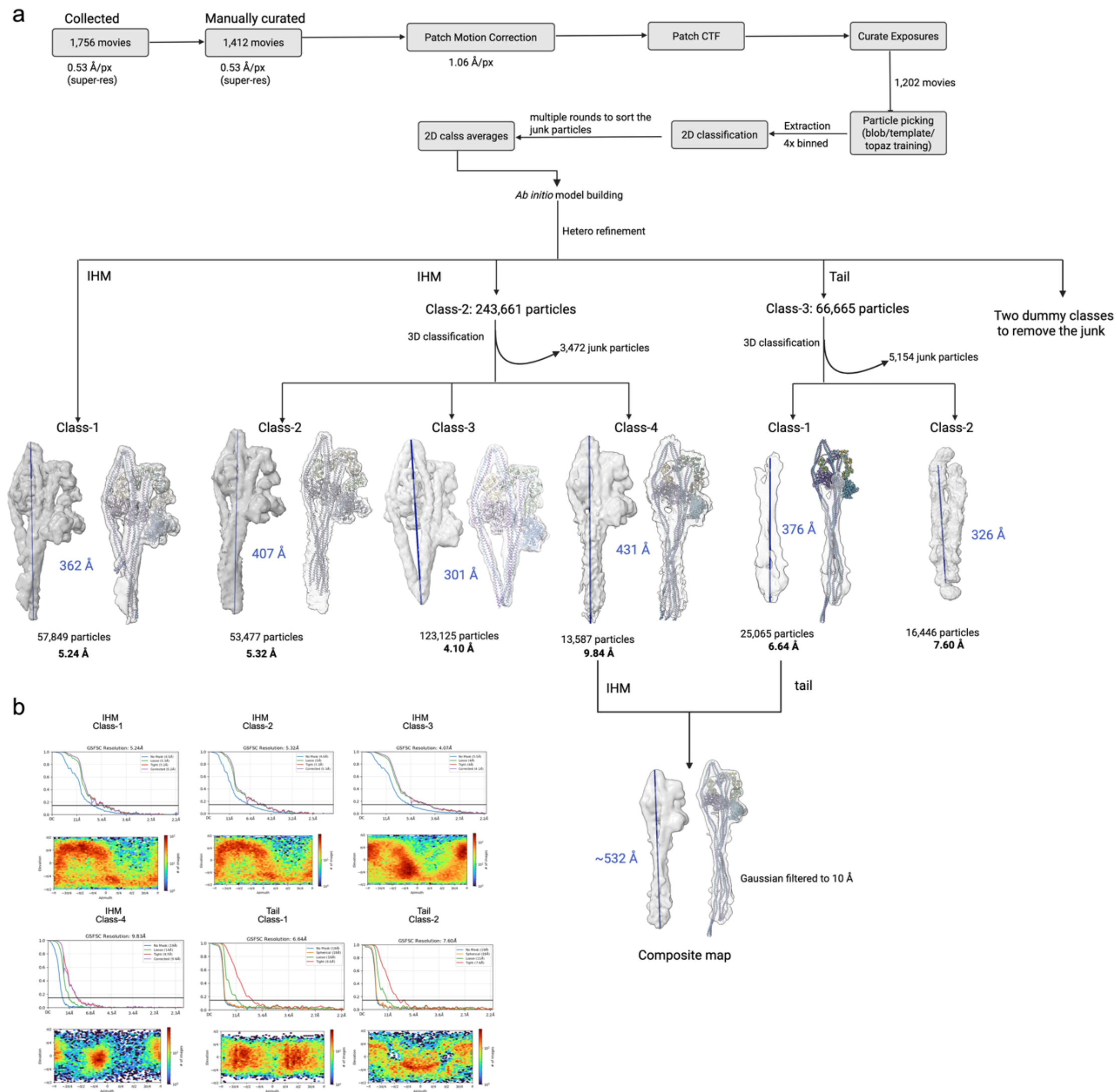

Supplementary Fig. 1: Cryo-EM workflow and conformational landscape of NM2B 10S.
a, The cryo-EM workflow revealed multiple conformations of the NM2B 10S molecule. Due to its elongated and flexible nature, particles from 2D class averages were extensively sorted following ab initio reconstruction using heterogeneous refinement. Class 1 displayed a unique orientation of segment 3 that bends away from segments 1 and 2 around the central coiled-coil region (CCR), with additional densities observed at the segment 1–2 junction. Class 2 particles were further divided into three 3D subclasses showing distinct orientations of segments 1 and 3 and variable CCR positioning. The Y-shaped tail regions were further classified into two additional classes. Classes 4 and 5 were combined to generate the composite map representing the full-length 10S structure. Blue line from the tip of the IHM along the spine of 10S is shown to indicate the resolved length of 10S structures and the length is indicated in blue.
b, FSCs and corresponding angular distribution of plots for all interacting heads motif (IHM) and tail classes are shown.

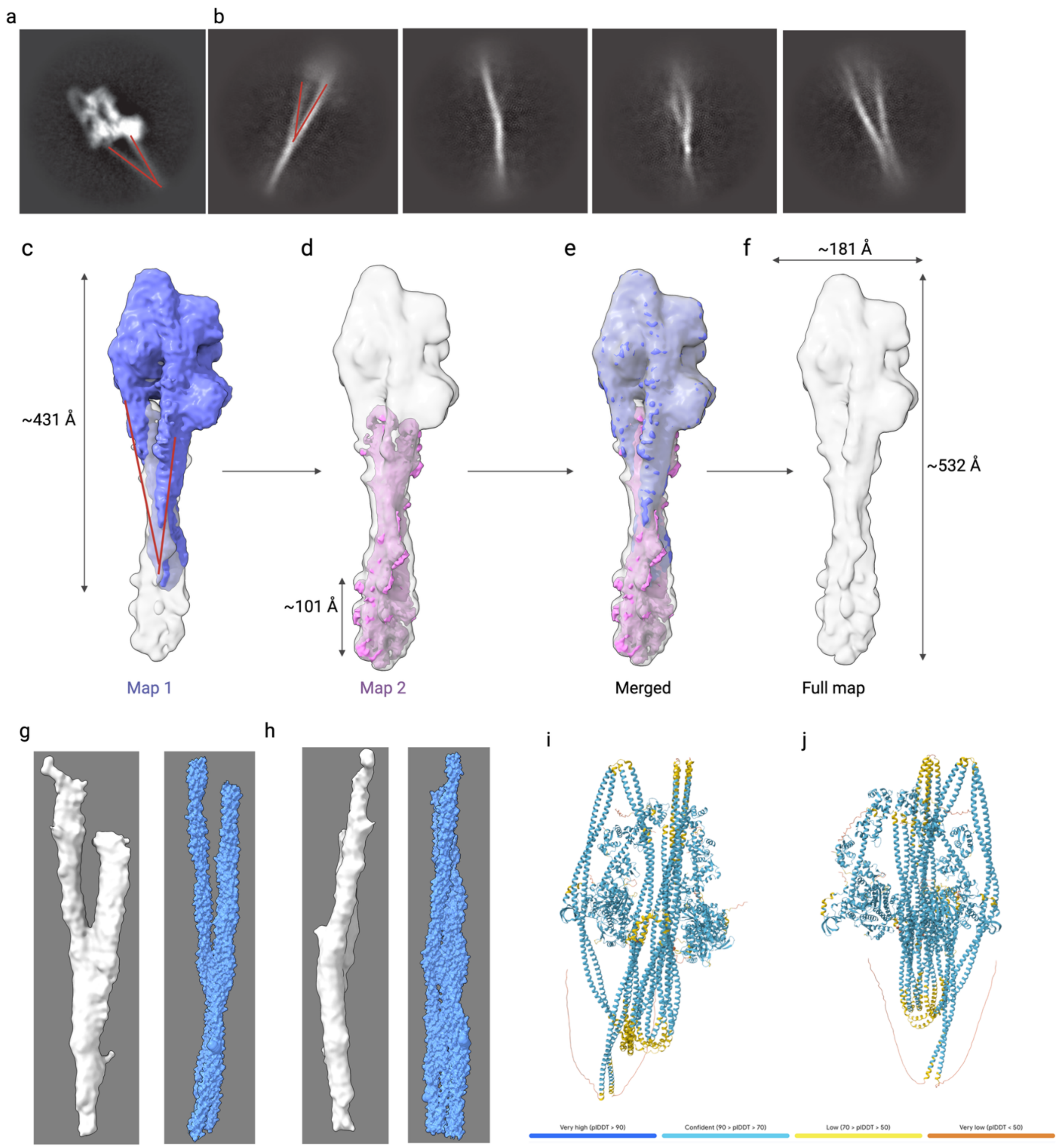

Supplementary Fig. 2: Reconstruction of full-length NM2B in the 10S conformation and conformational variability in the tail.

a, Representative 2D class average of the NM2B 10S IHM region, highlighting the proximal V-shaped tail portion.

b, 2D class averages of the NM2B tail region in various orientations. The V-shaped region proximal to the IHM is indicated with red lines.

c, 3D reconstruction of the IHM (blue, ~431 Å) superimposed onto the combined full-length map in white color.
d, 3D reconstruction of the distal tail region (pink, ~101 Å) superimposed onto the full-length map in white color.
e, Superposition of the IHM and distal tail region onto the full-length NM2B map.
f, Final composite full-length map of NM2B.
g, Front view of the 3D reconstructed cryo-EM map and corresponding atomic model of the tail.
h, Side view of the 3D reconstructed cryo-EM map and corresponding model of the tail region shows a pronounced bend in the tail.
i, Front view of the AlphaFold predicted symmetric NM2B structure.
j, Rear view of the AlphaFold predicted symmetric NM2B structure. The predicted model (iPTM = 0.88; pTM = 0.47) is colored by per-residue confidence score (pLDDT). Only coiled-coil regions, identified from the predicted structure, were used as rigid bodies for docking into the cryo-EM density map of NM2B.

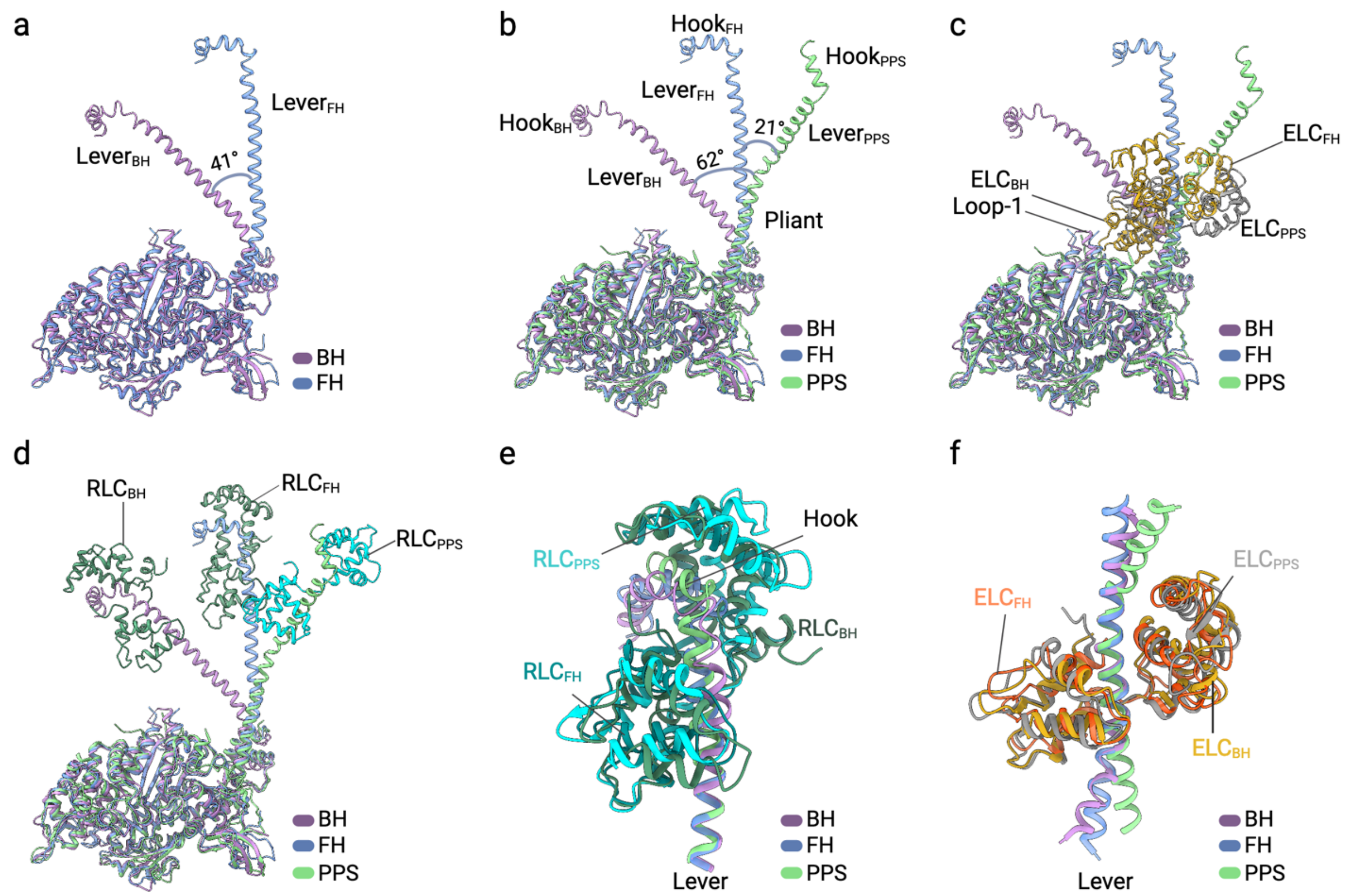

Supplementary Fig. 3: Conformation of the lever arm in the NM2B 10S structure.

a, Superimposition of the motor domains from the BH and FH of NM2B 10S. The angle between the BH and FH lever arms is ~ 41°.

b, Superimposition of the motor domains from BH and FH of NM2B 10S with the PPS structure of smooth muscle myosin-2 (PDB ID: 1QVI). The hook region at the end of the lever arm is also shown. The angle between the BH and PPS lever arms is ~ 62° , the angle between FH and PPS is ~ 21°.

c, Superimposition of the motor domains from BH and FH of NM2B 10S and scallop smooth muscle myosin-2 (PDB ID: 1QVI) including the associated ELCs. ELCs from BH and FH are colored golden yellow, and the ELC from the PPS structure is shown in gray.

d, Relative positioning of the RLCs in BH and FH of NM2B 10S and the canonical PPS structure (PDB ID: 1QVI).

e, Close-up view of the superimposed RLCs from BH (dark green) and FH (light green) of NM2B 10S and the PPS structure (PDB ID: 1QVI, cyan). The distal portion of the lever arm, including the hook region, was used for superposition.

f, Close-up view of the superimposed ELCs from BH (golden yellow) and FH (orange) of NM2B 10S and the PPS structure (PDB ID: 1QVI, gray). The proximal portion of the lever arm where the ELC binds was used for superposition.

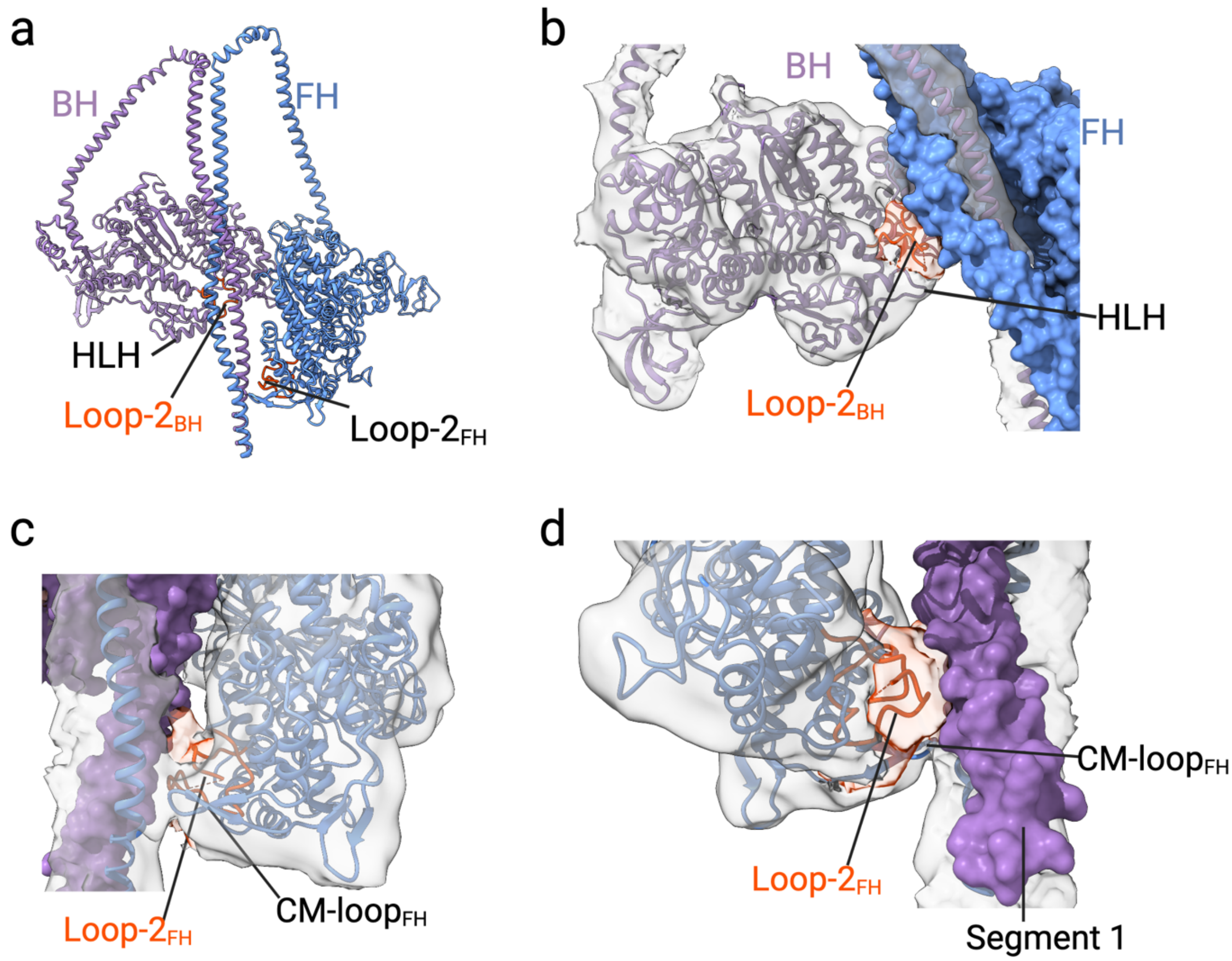

Supplementary Fig. 4: Sequestration of loop-2 weakens the interaction with actin.
a, Asymmetric arrangements of the heads in the NM2B 10S structure. Loop-2 regions in BH and FH are indicated as loop-2_{BH} and loop-2_{FH}, respectively. Loop-2 is colored in orange.
b, Close-up view of loop-2_{BH} (orange) is shown. Loop-2_{BH} is tucked behind segment 1.
c, Close-up view of loop-2_{FH} (orange) and CM $loop_{FH}$ are shown.
d, Rear view of loop-2_{FH} (orange) and CM-$loop_{FH}$ are shown. Loop-2_{FH} is in proximity to segment 1 and is folded back to prevent actin binding.

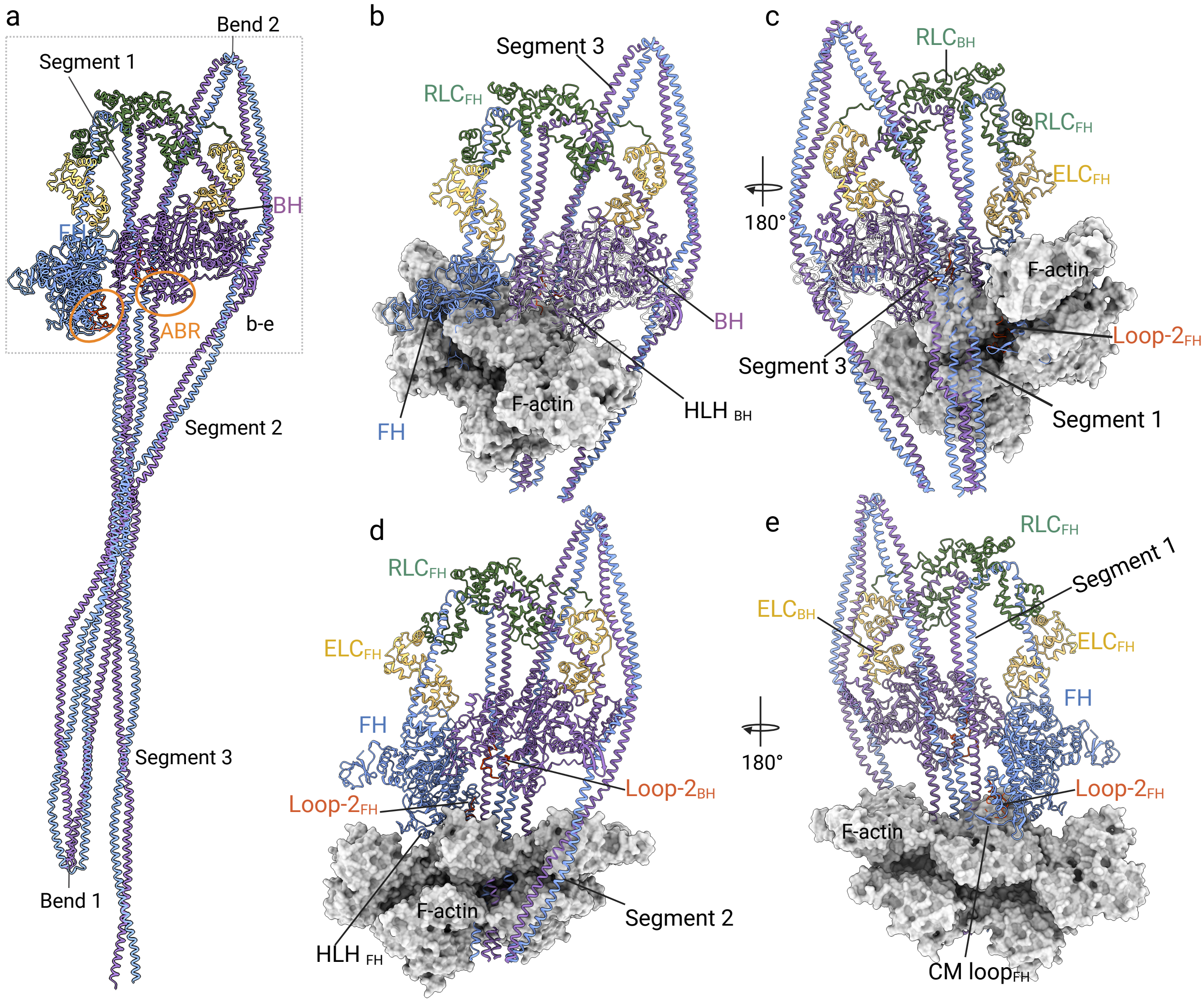

Supplementary Fig. 5: The 10S conformation sterically hinders actin binding.

a, Overview of full-length NM2B in the 10S conformation with the BH, FH, and tail segments indicated. The actin binding region (ABR) in each head is highlighted. Loop-2 is colored in orange-red.

b, Superimposition of rigor actin-myosin complex (PDB ID 5JLH) onto the block head shows that key actin binding regions including HLH, loop-2, and the lower 50kDa regions sterically obstruct the interaction with actin.

c, A rear view of panel **b** shows that the BH and all three tail segments sterically hinder actin binding.

d, Superimposition of rigor actin-myosin complex (PDB ID 5JLH) onto the 10S free head shows that key actin binding regions including HLH, CM-loop and loop-2 sterically obstruct the interaction with actin.

e, A rear view of panel **d** shows that the FH and all three tail segments sterically hinder actin binding along with the loop-2, the HLH motif, and the upper 50kDa in the FH prevent the interaction with actin.

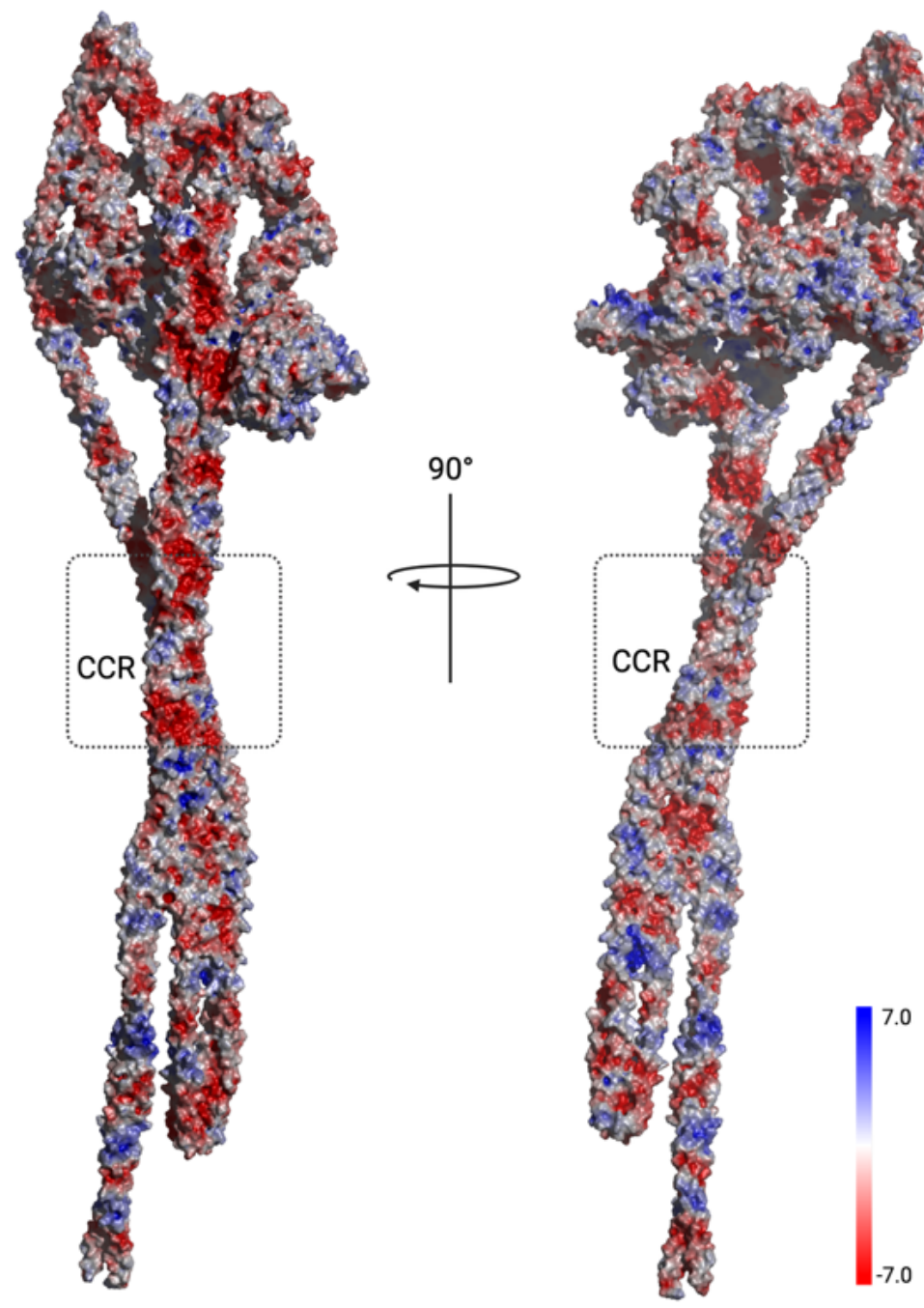

Supplementary Fig. 6: Electrostatic surface of NM2B 10S.
The electrostatic surface of the full-length NM2B 10S structure is shown, with negatively charged regions shown in red and positively charged regions shown in blue. The CCR is highlighted and shows alternating patches of positive and negative charge density.

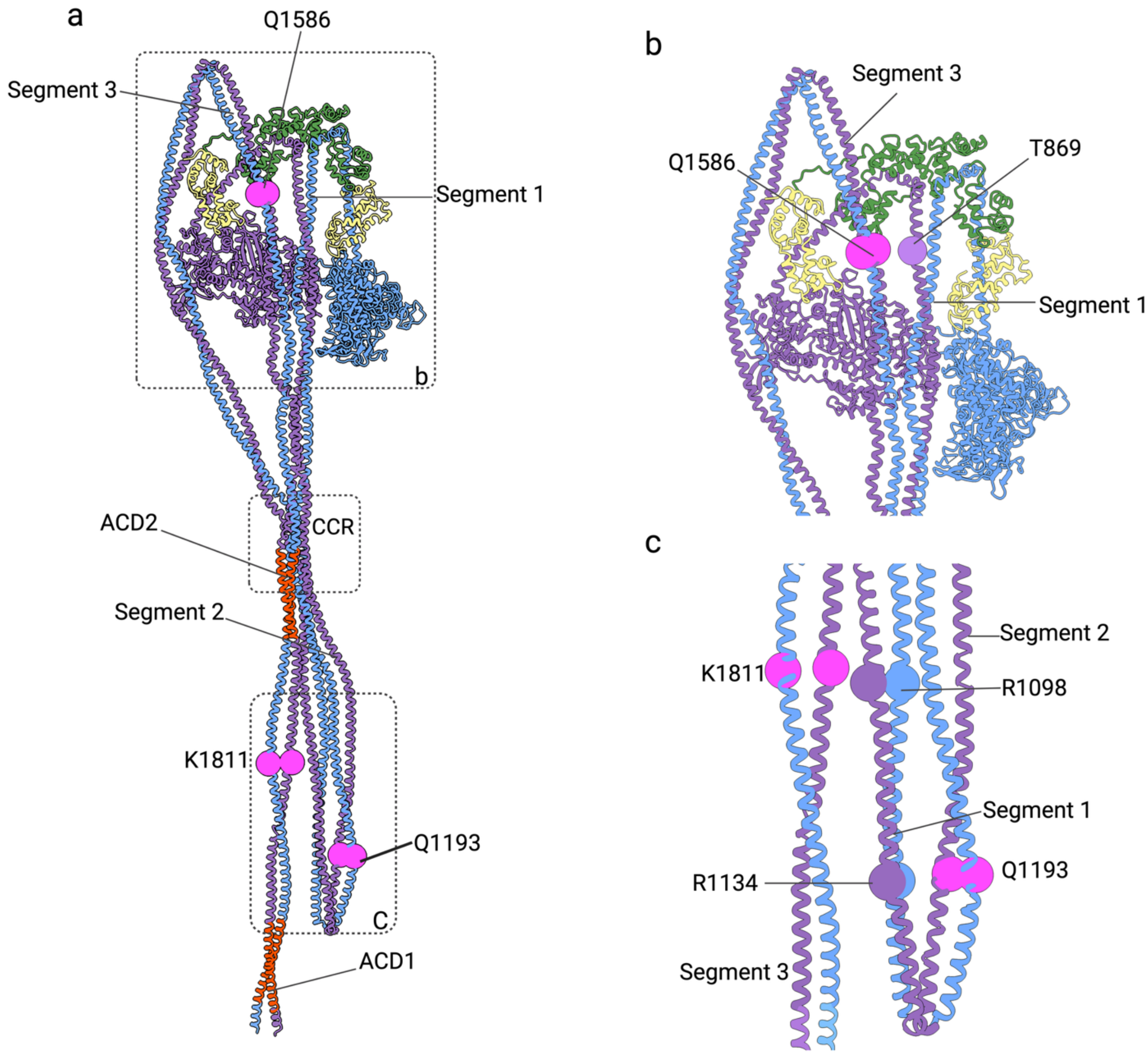

Supplementary Fig. 7: Location of skip residues and assembly competent domains in the full-length NM2B structure.
a, Three skip residues in the NM2B are highlighted in the full-length structure. The first skip residue Q1193 is located in segment 2 near the bend 1 region. Assembly competent domains (ACD) 1 and 2 are highlighted in orange. ACD2 overlaps with the CCR region. ACD1 is located at the distal end of segment 3. **b**, The second skip residue Q1586 is located in segment 3 near the bend 2 region. **c**, The third skip residue K1811 is located at the distal end of segment 3.

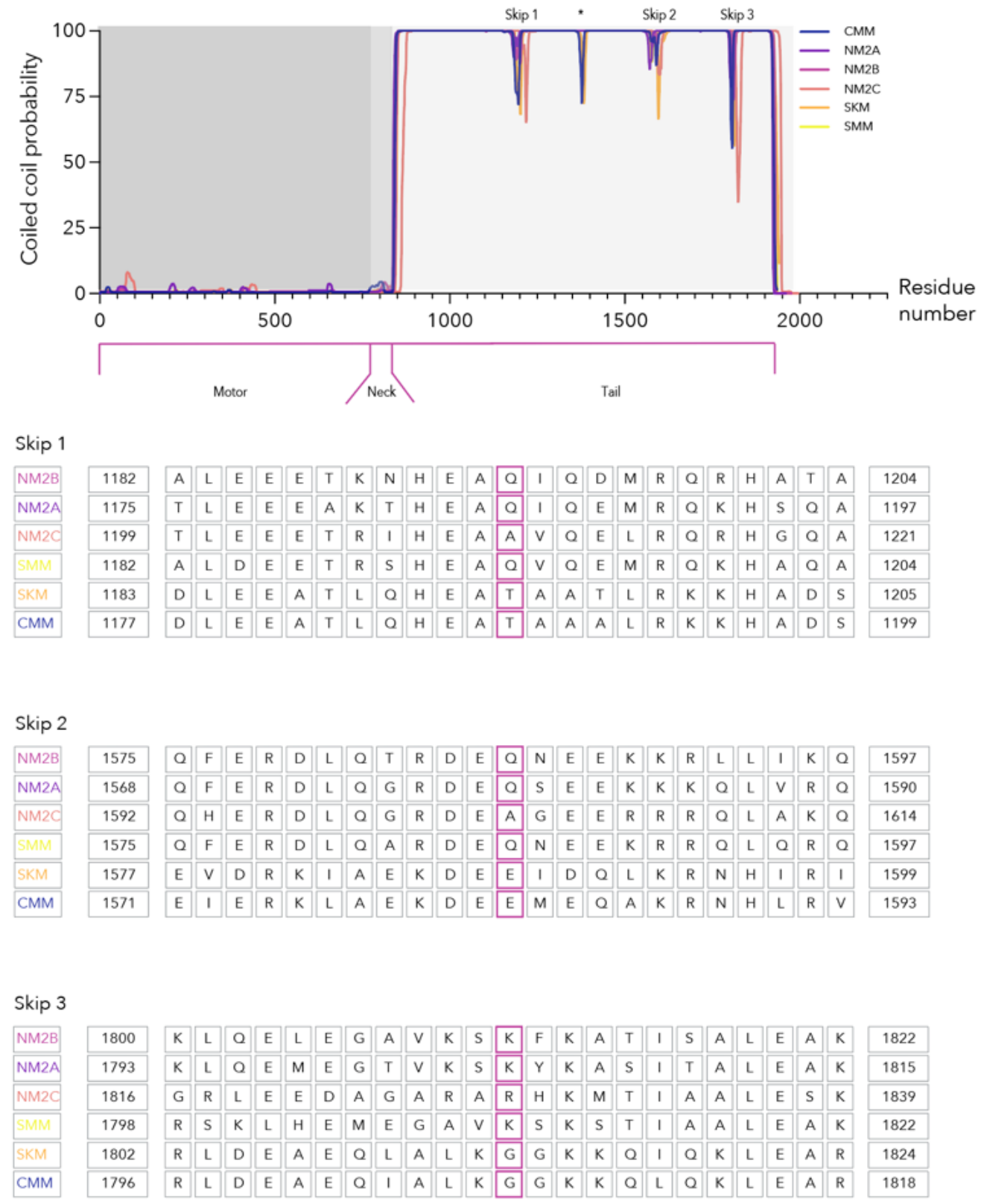

Supplementary Fig. 8: Location of skip residues in human myosin-2 heavy chains.

a, Coiled coil probability in human myosin-2 heavy chains. The position of skip residues is indicated. The asterisk denotes a skip residue that is absent in smooth and nonmuscle myosin-2 paralogs. CMM: β-cardiac myosin-2; NM2A: nonmuscle myosin-2A; NM2B: nonmuscle myosin-2B; NM2C: nonmuscle myosin-2C; SKM: skeletal muscle myosin-2; SMM: Smooth muscle myosin-2.

b, Sequence alignment of the skip 1 region. The skip residue is highlighted in pink.

c, Sequence alignment of the skip 2 region. The skip residue is highlighted in pink.

d, Sequence alignment of the skip 3 region. The skip residue is highlighted in pink.

Supplementary tables

Supplementary Table 1: Cryo-EM data collection, refinement, and validation statistics.

	Class-1: IHM-301Å EMD-73811 PDB 9Z4Q	Class-2: IHM-362Å EMD-73796 PDB 9Z3Z	Class-3 (EMD-73793) (PDB 9Z3W)	Composite Map (EMD-73797) (PDB 9Z40)	Class-4 IHM EMD-73798	Tail (class-1) EMD-73815	Tail (class-2) EMD-73818
**Data collection and processing**							
Magnification of K2 data	130,000x	130,000x	130,000x	130,000x	130,000x	130,000x	130,000x
Voltage (kV)	300 kV	300 kV	300 kV	300 kV	300 kV	300 kV	300 kV
Electron exposure ($e^-/Å^2$)	72	72	72	72	72	72	72
Defocus range (-μm)	1.0 to 3.5	1.0 to 3.5	1.0 to 3.5	1.0 to 3.5	1.0 to 3.5	1.0 to 3.5	1.0 to 3.5
Pixel size (Å)	0.53	0.53	0.53	0.53	0.53	0.53	0.53
Final particle images (no.)	123,125	57,849	53,477	N/A	13,874	25,065	16,446
Map resolution (Å)	4.10	5.24	5.34	~ 10Å	~9 Å	6.64 Å	7.60 Å
FSC threshold	0.143	0.143	0.143		0.143	0.143	0.143
Length of the 10S resolved (Å)	301	362	407	534	431	376	326
**Refinement**							
Initial model used (PDB code)	7MF3 and AlphaFold	7MF3 and AlphaFold	7MF3 and AlphaFold	7MF3 and AlphaFold			
Chains<br>Model resolution (Å)	6<br>4.37	6<br>5.70	6<br>7.06	6			
FSC threshold	0.143	0.143	0.143				
Map sharpening *B* factor ($Å^2$)	-107	-120	-125				
CC(mask)	0.80	0.78	0.69	0.59			
CC(box)	0.94	0.94	0.86	0.86			
Model composition<br>Non-hydrogen atoms<br>Protein residues<br>Ligands	 27538<br>3381<br>6	 28278<br>3470<br>6	 29378<br>3605<br>6	 35632<br>4380<br>6			
B factors ($Å^2$) (mean)<br>Protein<br>Ligand	 306.95<br>208.49	 343.43<br>265.21	 543.75<br>520.04	 558<br>599			
R.m.s. deviations<br>Bond lengths (Å)<br>Bond angles (°)	 <br>0.003<br>0.549	 0.004<br>0.620	 0.004<br>0.597	 0.004<br>0.694			
Validation<br>MolProbity score<br>Clashscore<br>Poor rotamers (%)	 1.49<br>9.16<br>0.03	 1.29<br>5.37<br>0.07	 1.23<br>4.61<br>0.98	 1.52<br>8.77<br>1.17			
Ramachandran plot<br>Favored (%)<br>Allowed (%)<br>Disallowed (%)	 99.23<br>0.71<br>0.06	 98.99<br>0.96<br>0.06	 99.22<br>0.78<br>0	 99.13<br>0.85<br>0.02			

Supplementary Table 2

Position of mutation (consensus sequence)	Position of mutation (original numbering)	Protein domain	Phenotypic effect, disease	Remarks	Reference
W37C	W37C	Motor	Neurodevelopmental	Missense	[73]
D74N	D74N	Motor	Marcus Gunn jaw-winking synkinesis without congenital ptosis	Missense	[74]
Y255C		Motor	Autism spectrum disorder	Missense	[75]
R270C	R280C	Motor	Neurodevelopmental	Missense	[76]
G313V	G313V	Motor	Congenital fibrosis of the extraocular muscles	Missense	[74]
N312S	N322S	Motor	Congenital fibrosis of the extraocular muscles	Missense	[74]
L385I	L395I	Motor	Neurodevelopmental	Missense	[73]
Q484E		Motor	Schizophrenia	Missense	[73]
G648D	G679D	Motor	Neurodevelopmental	Missense	[73]
G703D	G734D	Motor	Neurodevelopmental	Missense	[73]
E706K	E737K	Motor	Neurodevelopmental	Missense	[73]
R709Q	R740Q	Motor	Neurodevelopmental	Missense	[73]
G743S	G774S	Motor	Neurodevelopmental	Missense	[73]
R821Q	R852Q	Neck	Neurodevelopmental	Missense	[73]
R821W	R852W	Neck	Neurodevelopmental	Missense	[73]
R836X	R867X	Tail	Fetal hydrocephalus with ventriculo-megaly and aqueductal stenosis	Nonsense	[77]
E908X	E908X	Tail	Neurologic and developmental defects	Nonsense	[78,79]
E1280Q	E1280Q	Tail	Intellectual disability	Missense	[80]
R1471P	R1502P	Tail	Coloboma and ptosis	Missense	[81]
R1704Q	R1735Q	Tail	Marcus Gunn jaw-winking synkinesis with congenital ptosis	Missense	[74]
E1707K	E1738K	Tail	Congenital heart disease	Missense	[82]
E1709G	E1740G	Tail	Neurodevelopmental	Missense	[73]
K1809R	K1840R	Tail	Neurodevelopmental	Missense	[73]
R1944H	R1975H	Tail	Marcus Gunn jaw-winking synkinesis with congenital ptosis	Missense	[74]

Supplementary Table 2: Disease-associated missense mutations in human NM2B. Positions of mutations are given according to the protein consensus sequence (Uniprot ID: P35580). Published mutation data based on different isoform numbering were renumbered to match the consensus sequence.

Supplementary Movies

Supplementary movie 1: Maps and model of NM2B in the 10S conformation.

Supplementary movie 2: Differences in the head domains in NM2B in the 10S conformation. Superimposition of the BH (purple), FH (blue), and pre-powerstroke state structures (green, PDB ID: 1QVI). A morph from the FH to the BH state is shown in red. Light chains are not shown.

Supplementary movie 3: Comparison of electrostatic surfaces of Ser19 in the blocked-head RLC in its unphosphorylated and phosphorylated states.

Supplementary movie 4: Distribution of the electrostatic potential across NM2B in the 10S conformation. Negative charges are shown in red, positive charges are shown in blue.

Supplementary movie 5: Overall architecture of 10S. The FH and BH heavy chains are shown in blue and purple, respectively. The ELCs are shown in yellow and the RLCs in green. The segment orientation and arrangements from IHM towards the tail region are shown.

Supplementary movie 6: Actin-binding cleft is open in 10S structures. Superimposition of the BH (purple), FH (blue), rigor (gold, PDB ID: 5JLH), and ATP-bound (grey, PDB ID: 1MMN) motor domain structures. Actin-binding cleft is shown.

Supplementary movie 7: Location of skip residues in NM2B in the 10S conformation. The FH and BH heavy chains are shown in blue and purple, respectively. The ELCs are shown in yellow and the RLCs in green. Skip residues are shown as red spheres. ACD1 and 2 are shown in orange.

Supplementary movie 8: 3D variance analysis of NM2B in the 10S conformation.

Supplementary movie 9: Structural differences between the IHMs of NM2B and β-cardiac myosin-2. NM2B is shown in blue, β-cardiac myosin-2 in yellow. Light chains are not shown.

Supplementary References

73. Holtz, A.M. et al. Heterozygous variants in MYH10 associated with neurodevelopmental disorders and congenital anomalies with evidence for primary cilia-dependent defects in Hedgehog signaling. *Genet Med* **24**, 2065-2078 (2022).
74. Jurgens, J.A. et al. Expanding the genetics and phenotypes of ocular congenital cranial dysinnervation disorders. *Genet Med* **27**, 101216 (2025).
75. Li, J. et al. Genes with de novo mutations are shared by four neuropsychiatric disorders discovered from NPdenovo database. *Mol Psychiatry* **21**, 290-7 (2016).
76. Hamdan, F.F. et al. De novo mutations in moderate or severe intellectual disability. *PLoS Genet* **10**, e1004772 (2014).
77. Petrovski, S. et al. Whole-exome sequencing in the evaluation of fetal structural anomalies: a prospective cohort study. *Lancet* **393**, 758-767 (2019).
78. Iglesias, A. et al. The usefulness of whole-exome sequencing in routine clinical practice. *Genet Med* **16**, 922-31 (2014).
79. Tuzovic, L. et al. A human de novo mutation in MYH10 phenocopies the loss of function mutation in mice. *Rare Dis* **1**, e26144 (2013).
80. Carraro, M. et al. Assessment of patient clinical descriptions and pathogenic variants from gene panel sequences in the CAGI-5 intellectual disability challenge. *Hum Mutat* **40**, 1330-1345 (2019).
81. Scheidecker, S. et al. Novel MYH10 heterozygous variants associated to a syndrome combining mainly ptosis and ocular coloboma expand the MYH10 related phenotypes. *Eur J Hum Genet* (2025).
82. Jin, S.C. et al. Contribution of rare inherited and de novo variants in 2,871 congenital heart disease probands. *Nat Genet* **49**, 1593-1601 (2017).
