# Supplementary material for: Structural basis of nonmuscle myosin-2 autoinhibition mechanisms
Source: Nat Commun. 2026 Jun 20;17:7796. doi: 10.1038/s41467-026-74674-w (PMC13438149; doi:10.1038/s41467-026-74674-w)
Supplement: Supplementary file 5 [file 41467_2026_74674_MOESM2_ESM.pdf]

Title: Supplementary movie 1

Description: Maps and model of NM2B in the 10S conformation.

Title: Supplementary movie 2: Differences in the head domains in NM2B in the 10S conformation.

Description: Superimposition of the BH (purple), FH (blue), and pre-powerstroke state structures (green, PDB ID: 1QVI). A morph from the FH to the BH state is shown in red. Light chains are not shown.

Title: Supplementary movie 3

Description: Comparison of electrostatic surfaces of Ser19 in the blocked-head RLC in its unphosphorylated and phosphorylated states.

Title: Supplementary movie 4

Description: Distribution of the electrostatic potential across NM2B in the 10S conformation. Negative charges are shown in red, positive charges are shown in blue.

Title: Supplementary movie 5

Description: Overall architecture of 10S. The FH and BH heavy chains are shown in blue and purple, respectively. The ELCs are shown in yellow and the RLCs in green. The segment orientation and arrangements from IHM towards the tail region are shown.

Title: Supplementary movie 6

Description: Actin-binding cleft is open in 10S structures. Superimposition of the BH (purple), FH (blue), rigor (gold, PDB ID: 5JLH), and ATP-bound (grey, PDB ID: 1MMN) motor domain structures. Actin-binding cleft is shown.

Title: Supplementary movie 7

Description: Location of skip residues in NM2B in the 10S conformation. The FH and BH heavy chains are shown in blue and purple, respectively. The ELCs are shown in yellow and the RLCs in green. Skip residues are shown as red spheres. ACD1 and 2 are shown in orange.

Title: Supplementary movie 8

Description: 3D variance analysis of NM2B in the 10S conformation.

Title: Supplementary movie 9

Description: Structural differences between the IHMs of NM2B and β-cardiac myosin-2. NM2B is shown in blue, β-cardiac myosin-2 in yellow. Light chains are not shown.
